# Proximity Labeling To Map Host-Pathogen Interactions at the Membrane of a Bacterium-Containing Vacuole in Chlamydia trachomatis-Infected Human Cells

**DOI:** 10.1128/IAI.00537-19

**Published:** 2019-10-18

**Authors:** Macy G. Olson, Ray E. Widner, Lisa M. Jorgenson, Alyssa Lawrence, Dragana Lagundzin, Nicholas T. Woods, Scot P. Ouellette, Elizabeth A. Rucks

**Affiliations:** a Department of Pathology and Microbiology, University of Nebraska Medical Center, Omaha, Nebraska, USA; b University of Nebraska Medical Center, High School Alliance, Omaha, Nebraska, USA; c The Mass Spectrometry and Proteomics Core Facility, University of Nebraska Medical Center, Omaha, Nebraska, USA; d Eppley Institute for Research in Cancer and Allied Diseases, Fred & Pamela Buffett Cancer Center, University of Nebraska Medical Center, Omaha, Nebraska, USA; Yale University School of Medicine

**Keywords:** APEX2, *Chlamydia trachomatis*, host-pathogen interactions, Inc proteins, LRRF1, inclusion membrane, proximity labeling

## Abstract

Many intracellular bacteria, including the obligate intracellular pathogen Chlamydia trachomatis, grow within a membrane-bound bacterium-containing vacuole (BCV). Secreted cytosolic effectors modulate host activity, but an understanding of the host-pathogen interactions that occur at the BCV membrane is limited by the difficulty in purifying membrane fractions from infected host cells. We used the ascorbate peroxidase (APEX2) proximity labeling system, which labels proximal proteins with biotin *in vivo*, to study the protein-protein interactions that occur at the chlamydial vacuolar, or inclusion, membrane. An *in vivo* understanding of the secreted chlamydial inclusion membrane protein (Inc) interactions (e.g., Inc-Inc and Inc-eukaryotic protein) and how these contribute to overall host-chlamydia interactions at this unique membrane is lacking. We hypothesize some Incs organize the inclusion membrane, whereas other Incs bind eukaryotic proteins to promote chlamydia-host interactions. To study this, Incs fused to APEX2 were expressed in C. trachomatis L2. Affinity purification-mass spectrometry (AP-MS) identified biotinylated proteins, which were analyzed for statistical significance using significance analysis of the interactome (SAINT). Broadly supporting both Inc-Inc and Inc-host interactions, our Inc-APEX2 constructs labeled Incs as well as known and previously unreported eukaryotic proteins localizing to the inclusion. We demonstrate, using bacterial two-hybrid and coimmunoprecipitation assays, that endogenous LRRFIP1 (LRRF1) is recruited to the inclusion by the Inc CT226. We further demonstrate interactions between CT226 and the Incs used in our study to reveal a model for inclusion membrane organization. Combined, our data highlight the utility of APEX2 to capture the complex *in vivo* protein-protein interactions at the chlamydial inclusion.

## Abstract (precis)

Many intracellular bacteria, including the obligate intracellular pathogen Chlamydia trachomatis, grow within a membrane-bound bacterium-containing vacuole (BCV). Secreted cytosolic effectors modulate host activity, but an understanding of the host-pathogen interactions that occur at the BCV membrane is limited by the difficulty in purifying membrane fractions from infected host cells.

## INTRODUCTION

Chlamydia trachomatis is the leading cause of bacterial sexually transmitted infections (1). In 2017, 1.7 million cases were reported in the United States, with the highest incidence of infection being in people ages 15 to 29 years (2). Approximately 75% of infections are asymptomatic, and prolonged infection in women can lead to pelvic inflammatory disease and ectopic pregnancy (1). Infections in men can cause urethritis, epididymitis, and prostatitis (3, 4). Asymptomatic infections likely occur due to the obligate intracellular nature of this pathogen and manipulation of host cell responses by chlamydial secreted effectors (1).

Chlamydiae are developmentally regulated pathogens that reside within a membrane-bound vacuole, called an inclusion. C. trachomatis has two developmental forms: the infectious elementary body (EB) and the noninfectious reticulate body (RB). The EB infects a host cell, differentiates into an RB, and develops within a membrane-bound vacuole, termed an inclusion. The inclusion is initially derived from the eukaryotic plasma membrane that engulfs the invading EB and forms a barrier between the host and the pathogen (1, 5). Within the first few hours of infection, the chlamydial inclusion disassociates from the endosomal/lysosomal pathway. This process is likely mediated by the active modification by *Chlamydia* of the inclusion membrane via the insertion of type III secreted chlamydial inclusion membrane proteins (Incs) (6) and the recruitment of lipids and other host proteins to the chlamydial inclusion (7–15). Incs contain two or more hydrophobic transmembrane domains, with both termini being located on the host cytosolic face of the inclusion (5, 16–18). An estimated 50 to 70 *inc* genes (19) account for approximately 7% of the highly reduced chlamydial genome, indicating that these genes are important for optimal chlamydial development (20). In addition, Incs are temporally expressed throughout the developmental cycle (17, 21–23), which suggests that there are likely dedicated roles at specific points during the developmental cycle for individual Incs in the inclusion membrane.

To maximize the production of infectious EB progeny, C. trachomatis must recruit the necessary nutrients that it needs to develop yet protect against the host immune response. Given that the inclusion membrane is the host-pathogen interface and that chlamydiae extensively modify this membrane with secreted Incs, Inc proteins are likely central to achieve these functions. We hypothesize that Incs serve two functions: (i) to organize the inclusion membrane by forming nodes of interaction and spatially coordinating Inc-Inc interactions and (ii) to recruit eukaryotic proteins to facilitate necessary host-chlamydia interactions. Both functions are important to complete the developmental cycle and likely are not mutually exclusive. In support of this hypothesis, a previous bacterial adenylate cyclase two-hybrid (BACTH) study indicated that specific Inc proteins (e.g., IncF) bind multiple Incs (23), while other Incs (e.g., IncA) have been shown to interact with eukaryotic proteins (9, 12, 14, 24–26). Previous work has shown that knocking out certain Incs results in a weakened inclusion membrane and premature lysis (27). Although Incs represent the vast majority of identified chlamydial type III secreted proteins, little is known about their function in the inclusion membrane. This is largely due to the inherent difficulties of purifying Incs, which contain large hydrophobic regions (5), where the conditions required for solubilization do not preserve protein-protein interactions. By identifying the totality of protein binding partners for Incs during chlamydial infection, the function of specific Inc-protein interactions at the inclusion can be more completely understood.

Until recently, C. trachomatis was genetically intractable, which had been a major hindrance in advancing C. trachomatis-host interaction research. In the past, *in vitro* methods were used to identify Inc-protein binding partners. Such methods included transient transfection of epitope-tagged chlamydial Incs in uninfected host cells or the running of whole-cell uninfected lysates over a column bound by the soluble domain of a recombinant Inc protein (24). One drawback of ectopic expression is that the Inc proteins are expressed in the host cell out of their normal spatial context (e.g., they tend to aggregate in micelle-type structures) (28), increasing the possibility of identifying false interactions. Moreover, the ectopic expression of Inc proteins in eukaryotic cells, in contrast to their type III secretion from chlamydiae, is unlikely to result in correct protein folding and subcellular localization (i.e., in the inclusion membrane). The use of a recombinant Inc bound to a column and exposed to total eukaryotic cell lysate can promote false-positive interactions between eukaryotic proteins that are in subcellular compartments that do not typically interact with the chlamydial inclusion. An alternative strategy to purify chlamydial inclusions from large numbers of infected host cells requires lysis conditions and density gradient purification steps that can disrupt transient protein-protein interactions at the inclusion membrane and often results in lysis of the fragile inclusion membrane, yielding a total recovery rate of only about 8% (29). Furthermore, this purification method is labor-intensive and requires equipment that might not be available to laboratories other than the one in which this method was performed (29). In addition, and equally importantly, such methods do not capture potential Inc-Inc interactions. Not surprisingly, the Inc-protein binding partners identified to date using these methods are eukaryotic proteins. For example, IncG has been shown to bind the eukaryotic protein 14-3-3β to modify host signaling (9), IncD binds ceramide transfer protein (CERT) to acquire lipids (12), and IncE binds sorting nexin 5 (SNX5) and SNX6 to interfere with cargo trafficking (24, 25).

To capture dyanamic *in vivo* protein-protein interactions, including Inc-Inc interactions at the chlamydial inclusion, we were the first to use and characterize the feasibility, including important caveats, of using the ascorbate peroxidase (APEX2) proximity labeling system to identify Inc binding partners in the context of C. trachomatis infection (30). APEX2 has also recently been utilized by others in the field (with noted differences described in Discussion [31]). APEX2, a mutated soybean peroxidase (32, 33), can be fused to a protein of interest and activated during a short (1-min) reaction to covalently modify proximal proteins with a biotin molecule (33). This system can be used to capture *in vivo* snapshots of the dynamic protein-protein interactions that occur at the chlamydial inclusion during development. Incs fused to APEX2 are secreted by C. trachomatis and inserted in the inclusion membrane (30). Proteins proximal to the expressed Inc-APEX2 fusion protein are covalently modified with biotin after the addition of biotin-phenol and hydrogen peroxide to catalyze the APEX2 biotinylation reaction (30). An additional advantage of using APEX2 to identify Inc-protein binding partners is the ability to use high concentrations of detergent to solubilize hydrophobic membrane proteins, like Incs, because there is no need to maintain the binding partners after the covalent addition of biotin to neighboring proteins (30). Subsequently, the cells are lysed and the biotinylated proteins are affinity purified using streptavidin (Strep) beads and identified using affinity purification (AP)-mass spectrometry (MS).

We applied APEX2 to test our hypothesis using two Incs, IncF and IncA. IncF may be primarily involved in organizing the inclusion because it has been shown to interact extensively with other Incs via BACTH studies (23) and is expressed early after infection (22). IncA may primarily interact with eukaryotic proteins, as it contains a eukaryotic SNARE-like domain (34) and has been shown to bind fewer Incs by the same BACTH studies (23). We also created a truncated IncA (consisting of the IncA transmembrane domain [IncA_TM_]) to interrogate if removal of the C-terminal domain of IncA would alter the specificity of proteins labeled with this construct (30). Using the APEX2 proximity labeling system, we tested these interactions *in vivo* by transforming C. trachomatis serovar L2 cells with Inc-APEX2 fusion constructs that localize to the inclusion membrane when expressed (30). These experiments have helped define novel Inc-protein binding partners and whether Incs collaborate to support chlamydial development within the inclusion.

We carefully designed our experiments to (i) inducibly express Incs which localize in a pattern that resembles their endogenous form in an effort to detect protein-protein interactions under the most natural conditions, (ii) control for background contaminant proteins, and (iii) statistically analyze the mass spectrometry interaction data in an unbiased manner to determine the probability of a true protein interaction. In regards to the last point, our AP-MS data were analyzed for statistical significance using a Bayesian-based statistical analysis tool, significance analysis of the interactome (SAINT) (35). In each Inc-APEX2 data set, we identified chlamydial Inc proteins that were statistically significant, and we also identified eukaryotic proteins that had previously been shown to localize with the chlamydial inclusion. Importantly, we identified previously undescribed eukaryotic proteins at the inclusion membrane. Leucine-rich repeat flightless-interacting protein 1 (LRRF1) was identified in all of our Inc-APEX2 data sets and has been identified in other AP-MS studies (24, 29, 31). We also identified an LRRF1 binding partner, protein flightless 1 homolog (FLII), in our IncA-APEX2 data set, indicating that we were also identifying partial signaling pathways.

The presence of LRRF1 in our data sets gave us the opportunity to ask why this is a prominently identified protein in our and other AP-MS studies. We were skeptical that one protein was a true interactor with every single one of our Inc-APEX2 constructs. Therefore, we designed a series of experiments to help us understand how LRRF1 was identified through either a direct interaction with one of our Inc-APEX2 constructs or an interaction with an adjacent Inc but within the labeling radius of our Inc-APEX2 constructs. For the first time, we demonstrate that endogenous LRRF1 and FLII localize with the chlamydial inclusion. LRRF1 localization with the inclusion was conserved between closely related C. trachomatis serovars and strains. By the bacterial adenylate cyclase two-hybrid assay, LRRF1 was found to interact with the Inc CT226, which is consistent with the findings of a previous study which identified LRRF1 and FLII by transfecting Strep-tagged CT226 into uninfected eukaryotic cells (24). We also performed coimmunoprecipitation using CT226 fused to a FLAG tag (CT226_FLAG_) expressed from C. trachomatis and identified LRRF1 in the eluate. Overall, our proximity labeling system has identified both known and previously unreported proteins at the inclusion membrane and highlights the utility of an *in vivo* proximity labeling system to identify protein-protein interactions and how proteins are recruited to the chlamydial inclusion membrane.

## RESULTS

### Biotinylation of proximal proteins at the inclusion membrane using C. trachomatis L2 Inc-APEX2 transformants.

**FIG 1:**
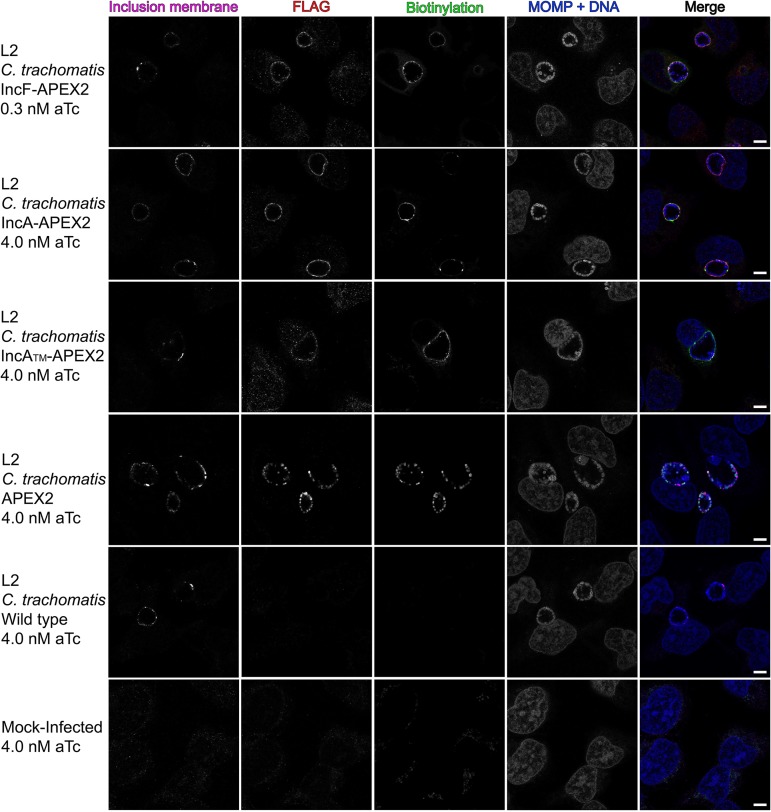
Localization and biotinylation of proteins proximal to the inclusion membrane in HeLa cells infected with C. trachomatis L2 transformants expressing Inc-APEX2 constructs. Coverslips were placed in two wells of a 6-well tissue culture plate to ensure appropriate biotinylation. HeLa cells that were infected with C. trachomatis serovar L2 transformed with the indicated APEX2 constructs or the C. trachomatis L2 wild type (WT) or that were mock infected were induced for construct expression with the indicated concentrations of anhydrotetracycline (aTc) at 7 hpi. Biotin-phenol was added at 23.5 hpi and biotinylation was catalyzed at 24 hpi by the addition of 3 mM H_2_O_2_ for 1 min, after which the reaction was quenched. Coverslips were removed from the 6-well plate and processed for immunofluorescence to visualize biotinylated proteins (the streptavidin-488 conjugate), expression of the construct (anti-FLAG, red), chlamydiae (MOMP) and DNA (DAPI; blue), and the inclusion membrane (anti-CT223; pink). Coverslips were imaged using a Zeiss LSM 800 confocal microscope at a ×63 magnification with a ×2 zoom. Bars = 5 μm.

To examine our hypothesis that some Incs preferentially interact with other Inc proteins, whereas other Incs primarily interact with eukaryotic proteins, we used the ascorbate peroxidase (APEX2) proximity labeling system to determine chlamydial Inc binding partners *in vivo* (30). To do this, we transformed C. trachomatis serovar L2 with a plasmid encoding IncF-APEX2, the IncA transmembrane domain (IncA_TM_)-APEX2, IncA-APEX2, or APEX2 only controlled by an anhydrotetracycline (aTc)-inducible promoter system. IncF has previously been shown to interact with several Incs (23) and contains a short cytosolic domain, which could limit its ability to interact with eukaryotic proteins. In the same study, IncA interacted with fewer Incs (23) and was found to have a large cytosolic domain with a eukaryotic SNARE-like domain (34, 36–38), suggesting that IncA might preferentially interact with eukaryotic proteins. IncA_TM_-APEX2 is truncated to have a short cytosolic domain like IncF-APEX2 and is used to determine if the C terminus of IncA confers specificity toward determining protein binding partners (30). The construct with APEX2 only is a negative control included in each experiment and, when expressed in transformed C. trachomatis L2, remains in the bacterial cytosol because it lacks the type III secretion signal (Fig. 1) (30). All proximity labeling experiments were performed using a plaque-cloned population of C. trachomatis L2 transformants. HeLa 229 cells were infected with the C. trachomatis L2 IncF-APEX2, IncA_TM_-APEX2, IncA-APEX2, or APEX2 transformant and induced with anhydrotetracycline (aTc). As previously determined, this resulted in the expression and localization of each construct that matches endogenous IncA and IncF (21, 30, 39). An epitope tag (FLAG) was located at the N terminus of APEX2 and was used to visualize the localization of the various APEX2 constructs expressed from C. trachomatis L2.

For each biotinylation experiment, coverslips were placed in two wells of a six-well plate to confirm the presence of biotinylated proteins at the inclusion membrane by indirect immunofluorescence microscopy. This was performed for each of the test conditions and controls. HeLa cells were infected with C. trachomatis L2 IncF-APEX2, IncA_TM_-APEX2, IncA-APEX2, or APEX2, and construct expression was induced (with 0.3 nM aTc for IncF-APEX2 and 4 nM aTc for all other transformants) at 7 h postinfection (hpi). Biotin-phenol was added to each well at 23.5 hpi, and at 24 hpi, hydrogen peroxide (H_2_O_2_) was added to the wells to catalyze the biotinylation reaction during a 1-min incubation. After biotinylation, the enzymatic APEX2 activity was quenched. The coverslips were removed from the wells, fixed, and then stained for immunofluorescence to confirm appropriate biotinylation. Separately, the lysate was collected as indicated in Materials and Methods and processed after confirming biotinylation by indirect immunofluorescence. The expression of each construct containing APEX2 and biotinylation at the inclusion membrane was observed using each of the C. trachomatis Inc-APEX2 transformants (Fig. 1). For C. trachomatis L2 APEX2, which lacks a type III secretion signal, biotinylation was colocalized with the bacterial cytosol (Fig. 1). For C. trachomatis L2 wild-type (i.e., untransformed)-infected and mock-infected (i.e., no APEX2) HeLa cells, faint biotinylation was observed in subcellular structures consistent with mitochondria, but no biotinylation was detected at the inclusion of the C. trachomatis L2 wild type (Fig. 1). This confirmed that proteins proximal to the inclusion were biotinylated using the Inc-APEX2 constructs.

### Verification of Inc-APEX2 labeling activity on the cytosolic face of the inclusion membrane by electron microscopy.

C. trachomatis L2 transformed with IncF-APEX2, IncA_TM_-APEX2, and IncA-APEX2 targets the constructs to the inclusion membrane, with the C terminus (which contains APEX2) being exposed to the host cytosol (40). We used electron microscopy to provide further support for the finding that the C. trachomatis L2 Inc-APEX2 transformants labeled the cytosolic face of the chlamydial inclusion (41). For these studies, HeLa cells were infected with the wild type (i.e., untransformed chalmydiae) or the C. trachomatis L2 Inc-APEX2 transformant, and the monolayers were treated with aTc to induce APEX2 fusion protein expression. Then, the cells were fixed with a glutaraldehyde and paraformaldehyde solution, which maintains APEX2 activity (41), and labeled with or without 3,3′-diaminobenzidine (DAB). DAB and hydrogen peroxide (H_2_O_2_) diffuse into nonpermeabilized cells, and in the proximity of APEX2, DAB polymerizes (32, 41, 42). Upon polymerization, DAB becomes membrane impermeant and remains closely associated with the site of polymerization (41). DAB reacts with the heavy metal (osmium tetroxide) used in the staining procedure to create a contrast that can be observed by electron microscopy (41).

**FIG 2:**
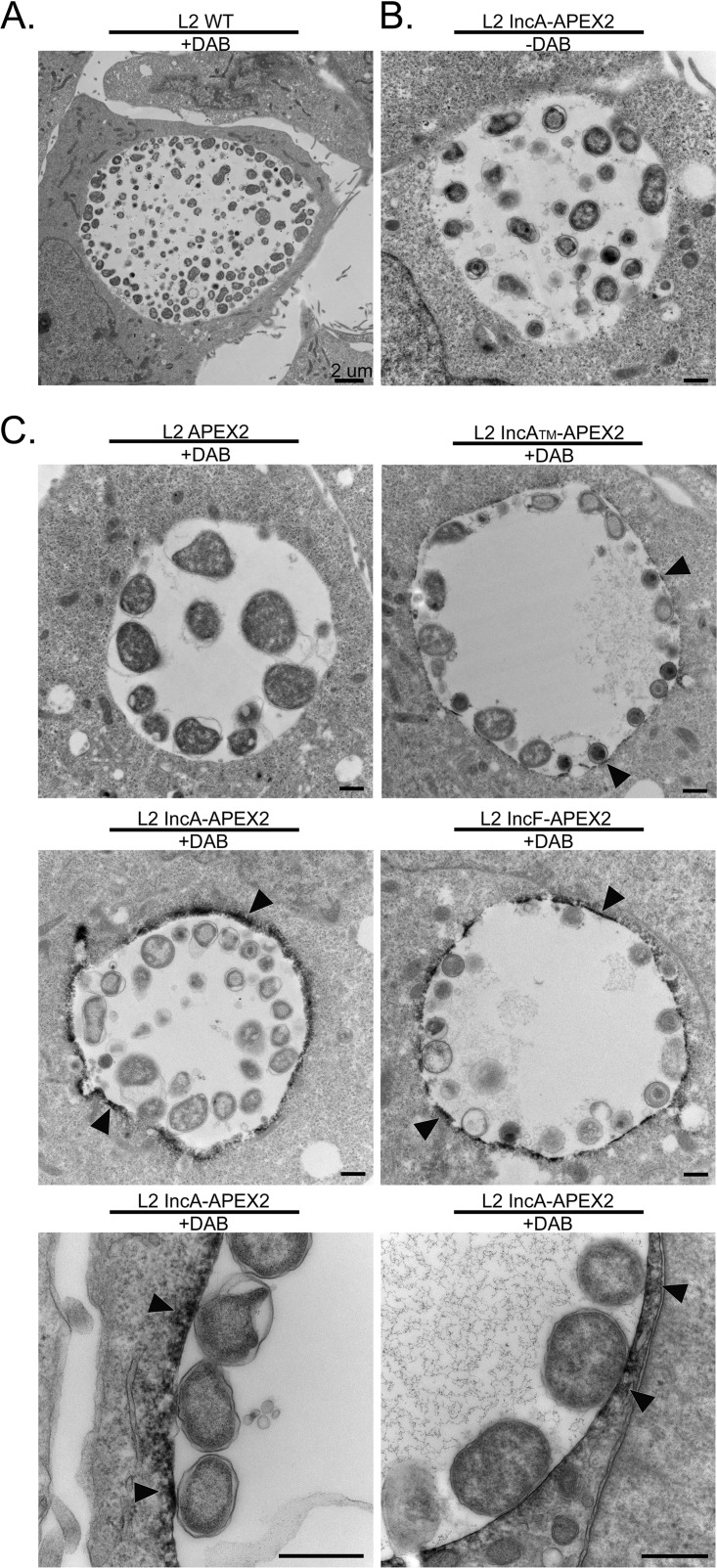
Ultrastructural localization of APEX2 activity to the cytosolic face of the inclusion membrane in HeLa cells infected with C. trachomatis L2 transformants expressing Inc-APEX2 constructs, as determined by electron microscopy. HeLa cells seeded onto electron microscopy-grade, cell culture-treated coverslips were infected with C. trachomatis serovar L2 transformed with the indicated constructs or C. trachomatis serovar L2 wild-type (WT) and induced with anhydrotetracycline (aTc) at 7 hpi (0.3 nM aTc for the IncF-APEX2 transformants and 5 nM aTc for all others). At 24 hpi, a glutaraldehyde and paraformaldehyde fixing solution was added to each sample and the samples were incubated on ice. Next, the samples were pretreated with DAB (or not, as indicated) 30 min prior to labeling by the addition of H_2_O_2_ solution (also containing DAB) to catalyze DAB polymerization. The reaction was quenched with glycine and processed for electron microscopy as indicated in Materials and Methods. (A) C. trachomatis L2 wild type (WT) treated with DAB; (B) C. trachomatis L2 IncA-APEX2 without DAB; (C) C. trachomatis L2 transformants treated with DAB. DAB polymer staining around the inclusion is indicated by arrowheads. Bars = 2 μm (A) and 500 nm (B and C).

As seen in Fig. 2A, no DAB polymerization was observed at the inclusion membrane in HeLa cells infected with wild-type C. trachomatis L2. To control for background activity, HeLa cells were infected with C. trachomatis L2 IncA-APEX2 and induced for expression but not treated with DAB (Fig. 2B). In these samples, no DAB staining was observed at the inclusion membrane (Fig. 2B). There was no detectable DAB labeling at the inclusion membrane in HeLa cells infected with C. trachomatis L2 APEX2, but we did not observe strong DAB polymerization within individual organisms (Fig. 2C). In HeLa cells infected with the C. trachomatis L2 IncF-APEX2, IncA_TM_-APEX2, or IncA-APEX2 transformant, DAB polymerization was observed at the inclusion membrane (Fig. 2C, arrowheads). However, there appeared to be less DAB labeling with IncA_TM_-APEX2 than with IncF-APEX2 and IncA-APEX2. Overall, by electron microscopy, we observed Inc-APEX2-directed DAB labeling at the inclusion membrane, and this labeling appeared on the cytosolic face of the inclusion membrane.

### Western blotting detection of APEX2-containing constructs expressed from C. trachomatis L2 transformants.

To confirm the correct expression of each construct containing APEX2, HeLa cells were infected with the C. trachomatis L2 APEX2, IncF-APEX2, IncA_TM_-APEX2, or IncA-APEX2 transformant and either not induced or induced (with 0.3 nM aTc for IncF-APEX2 and 5 nM aTc for all other transformants) at 7 hpi. Cell lysates were collected at 24 hpi and prepared for affinity purification using FLAG magnetic beads essentially as previously described (43). The eluates were blotted for the presence of each APEX2-containing construct using anti-FLAG antibody (the FLAG epitope tag is located at the N terminus of APEX2). Lower levels of IncF-APEX2 (39.7 kDa) than of IncA_TM_-APEX2 (40.7 kDa), IncA-APEX2 (59.3 kDa), and APEX2 (30.3 kDa) were detected (see Fig. S1 in the supplemental material). We detected some leaky expression of IncF-APEX2, IncA_TM_-APEX2, and IncA-APEX2 in our uninduced samples (Fig. S1). As a loading control, the solubilized lysate was blotted for the presence of chlamydial heat shock protein 60 (cHsp60) (Fig. S1, bottom; the cHsp60 antibody was a kind gift from Rick Morrison, University of Arkansas for Medical Sciences, Little Rock, AR). These data confirmed that each APEX2-containing construct was expressed from the C. trachomatis L2 transformants at the expected molecular weight.

### Affinity purification of biotinylated proteins.

After confirming the correct construct localization, labeling activity at the inclusion membrane, and molecular weights of the proteins expressed from C. trachomatis L2, the lysates from the C. trachomatis L2 IncF-APEX2, IncA_TM_-APEX2, and IncA-APEX2 transformants and the negative-control-infected HeLa cells from the biotinylation experiments described above (Fig. 1) were affinity purified to isolate biotinylated proteins. The negative controls, which consisted of mock-infected, C. trachomatis L2 wild-type-infected, and C. trachomatis L2 APEX2-infected HeLa cells treated with biotin-phenol and hydrogen peroxide (to catalyze labeling), served to control for the background, endogenous biotinylated proteins. As described previously, the major background endogenous biotinylated proteins included eukaryotic mitochondrial carboxylases (75 and 125 kDa) (30, 44, 95) and, in C. trachomatis L2-infected HeLa cells, the chlamydial biotin ligase (21 kDa), which uses biotin as a cofactor (30, 45). We did not include uninduced C. trachomatis L2 transformants in our analysis because we observed some leaky construct expression and were concerned that using them as a negative control would subtract true interacting proteins during the analysis step (Fig. S1).

**FIG 3:**
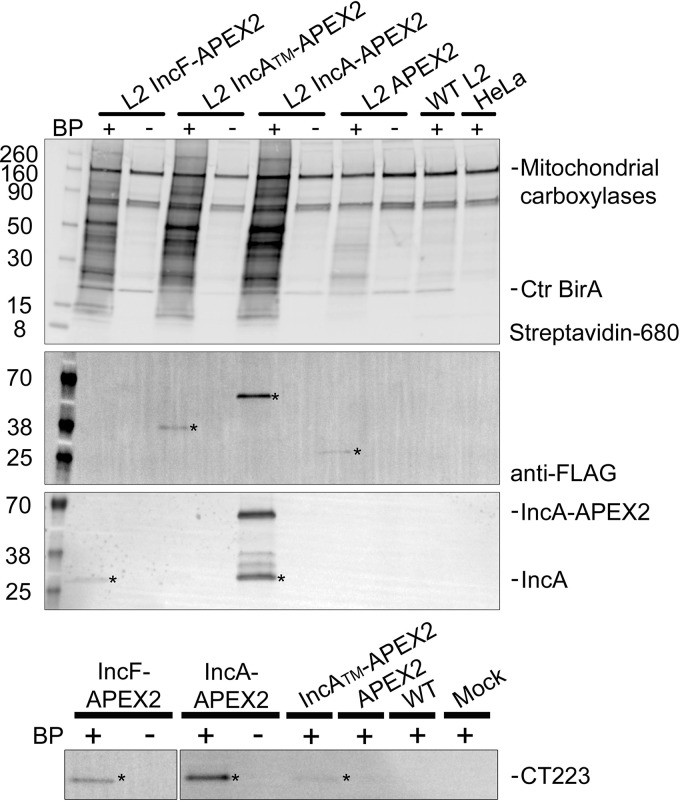
Western blot detection of affinity-purified biotinylated proteins. HeLa cells infected with C. trachomatis L2 Inc-APEX2 transformants or the wild type (WT) or mock-infected cells were induced with anhydrotetracycline (aTc) at 7 hpi (0.3 nM aTc for IncF-APEX2 transformants and 4 nM for all others). Biotin-phenol (BP) was added 30 min prior to the biotinylation reaction at 24 hpi. Biotinylation was catalyzed by the addition of 3 mM H_2_O_2_ for 1 min and stopped with a quenching wash solution. Biotinylated proteins were affinity purified from solubilized lysates using streptavidin beads, eluted in sample buffer, separated by SDS-PAGE, and transferred to a PVDF membrane for Western blotting. The eluate fraction was probed for biotinylated proteins (streptavidin-680 conjugate), construct expression (anti-FLAG antibody), IncA (anti-IncA antibody), and CT223 (anti-CT223 antibody) and imaged using an Azure c600 system. Asterisks indicate the detected proteins. Numbers on the left indicate molecular masses (in kilodaltons). See Fig. S1 in the supplemental material.

Biotinylated proteins were affinity purified using streptavidin beads and visualized by Western blotting using a fluorescent streptavidin conjugate (Fig. 3, Streptavidin-680 panel). Biotinylated proteins were detected from each of the C. trachomatis L2 IncF-APEX2, IncA-APEX2, and IncA_TM_-APEX2 transformants that received both biotin-phenol and H_2_O_2_ (Fig. 3, Streptavidin-680 panel). Without the addition of biotin-phenol to the C. trachomatis L2 Inc-APEX2 transformants, only endogenous biotinylated proteins were detected. Similarly, in each of the negative controls, C. trachomatis L2 APEX2-infected, C. trachomatis L2 wild-type-infected (not transformed), and mock-infected HeLa cells, only background endogenous biotinylated proteins were detected (Fig. 3, Streptavidin-680 panel).

To determine if the expressed constructs containing APEX2 were biotinylated *in vivo* and affinity purified, we blotted the eluates using an anti-FLAG antibody (APEX2 contains the FLAG epitope in the N terminus). We detected biotinylated IncA_TM_-APEX2 (40.7 kDa), IncA-APEX2 (59.3 kDa), and APEX2 (30.3 kDa) (Fig. 3, anti-FLAG panel). We did not observe biotinylated IncF-APEX2 (40.7 kDa) in the eluate fraction, which is likely a result of the lower expression necessary to preserve its correct localization (30) (Fig. S1). In addition, to determine if we could detect solubilized endogenous chlamydial Incs, we used an anti-IncA antibody (a gift from Ted Hackstadt, NIAID, Rocky Mountain Laboratories, Hamilton, MT) and an anti-CT223 antibody (a gift from R. Suchland, University of Washington, WA, and D. Rockey, Oregon State University, OR) to blot the eluates from the streptavidin affinity purification. We detected endogenous IncA in the eluate from C. trachomatis L2 IncF-APEX2 and IncA-APEX2 (Fig. 3, IncA panel). The IncA antibody is specific for the C terminus, so it detects IncA-APEX2 (the 59.3-kDa band) containing full-length IncA and not the truncated IncA_TM_-APEX2 construct, which lacks the epitope that is recognized by the antibody. We also detected CT223 (29.3 kDa) in the streptavidin affinity-purified eluate from each of the C. trachomatis L2 Inc-APEX2 samples but not in the negative controls (Fig. 3, CT223 panel). These Western blotting data provide an initial validation of our proximity labeling system because IncA homotypic interactions have been described previously (23, 34, 36, 37, 46) (e.g., IncA-APEX2 interacts with endogenous IncA in the inclusion membrane). These data are also consistent with previously published *in vivo* protein-protein interaction data obtained using the BACTH system, which identified IncF and IncA interactions (23).

### Mass spectrometry identification of streptavidin affinity-purified biotinylated C. trachomatis L2 and eukaryotic proteins.

To identify the proteins proximal to or interacting with the inclusion membrane that were biotinylated *in vivo* using the APEX2 proximity labeling system, the eluates from streptavidin affinity purification were briefly electrophoresed, sectioned, and then processed for mass spectrometry identification. To enhance peptide identification by mass spectrometry, individual gel sections were digested with two enzymes, trypsin and Asp-N (47), and then processed as indicated in Material and Methods. Five biological replicates for each condition were analyzed by tandem mass spectrometry (MS/MS), and individual peptides were identified by performing Mascot searches against the C. trachomatis L2 (strain 434/Bu) database and the Homo sapiens database. Our analysis detected 810 C. trachomatis L2 proteins (Table S1) and over 5,000 eukaryotic proteins (Table S2) in total from the combined data sets. To analyze our mass spectrometry data for statistical significance and to remove nonspecific or background biotinylated proteins, we used significance analysis of the interactome (SAINT) (35). SAINT uses quantitative data embedded in the raw mass spectrometry data from label-free quantification methods to filter out background peptides (35). The peptide spectrum for a protein (i.e., the prey) identified in the sample of interest (i.e., the bait) is normalized to both the protein length and the total number of spectra compared to those for the negative controls. Bayesian statistics are used to calculate the probability of an interaction between each bait-prey interaction identified. The calculated probability is expressed as the Bayesian false discovery rate (BFDR). We used a BFDR of less than or equal to 0.05 as a cutoff for our analysis parameters, which indicates the probability that the interaction is true (i.e., at a BFDR of 0.05, we are 95% confident in the protein associations described).

When we analyzed the C. trachomatis L2 proteins for statistical significance, several Inc proteins were among the top SAINT-identified significant hits obtained using our Inc-APEX2 constructs (Table 1; Table S1). Using our BFDR cutoff (BFDR ≤ 0.05), we identified three statistically significant chlamydial proteins using C. trachomatis L2 IncF-APEX2 and IncA-APEX2 and two significant proteins using C. trachomatis L2 IncA_TM_-APEX2. CT223 was the only chlamydial protein that was identified to be statistically significant using each C. trachomatis L2 Inc-APEX2 transformant. IncA was detected using C. trachomatis L2 IncA-APEX2 and IncA_TM_-APEX2. The identification of CT223 and IncA by mass spectrometry using IncA-APEX2 is supported by the detection of proteins eluted from the streptavidin affinity-purified lysate (Fig. 3). The statistically significant chlamydial proteins that were unique to the individual C. trachomatis L2 transformants included IncD and IncF, which were identified using C. trachomatis L2 IncF-APEX2, and outer membrane complex B (OmcB), which was identified using C. trachomatis L2 IncA-APEX2 (Table 1; Table S1). Additional chlamydial Inc proteins that were detected by mass spectrometry but that did not make the BFDR cutoff (BFDR ≤ 0.05) using C. trachomatis L2 IncA-APEX2 included IncC (BFDR = 0.09), CT813 (BFDR = 0.1), IncD (BFDR = 0.11), and IncE (BFDR = 0.2) (Table S1). In contrast, there were no additional Incs identified using C. trachomatis L2 IncA_TM_-APEX2 with a less stringent cutoff (BFDR ≤ 0.2). Using C. trachomatis L2 IncF-APEX2, IncA (BFDR = 0.12), CT228 (BFDR = 0.15), and IncE (BFDR = 0.18) were detected (Table S1). Although IncA was not statistically significant when IncF-APEX2 was used, IncA was detected in the affinity-purified eluate of IncF-APEX2 by Western blotting (Fig. 3; Table S1). These data are also supported by previously observed IncF-IncA interactions by BACTH (23) and IncA-IncA interactions that have been previously described (23, 36). Importantly, our AP-MS data analyzed against the C. trachomatis L2 (434/Bu) database were supported by our Western blotting data.

**TABLE 1:** Significant C. trachomatis L2 proteins

Sample	UniProt identifier		Protein name^*a*^	BFDR^*b*^
	Protein	Gene name		
IncF-APEX2	A0A0H3MKT3_CHLT2	CTL0476	CT223	0
	INCD_CHLT2	CTL0370	IncD	0.02
	INCF_CHLT2	CTL0372	IncF	0.03
IncA_TM_-APEX2	A0A0H3MD02_CHLT2	CTL0374	IncA	0
	A0A0H3MKT3_CHLT2	CTL0476	CT223	0.02
IncA-APEX2	OMCB_CHLT2	CTL0702	OmcB	0
	A0A0H3MKT3_CHLT2	CTL0476	CT223	0
	A0A0H3MD02_CHLT2	CTL0374	IncA	0

a The protein name is indicated using the naming convention for C. trachomatis serovar D.

b BFDR, SAINT Bayesian false discovery rate.

**FIG 4:**
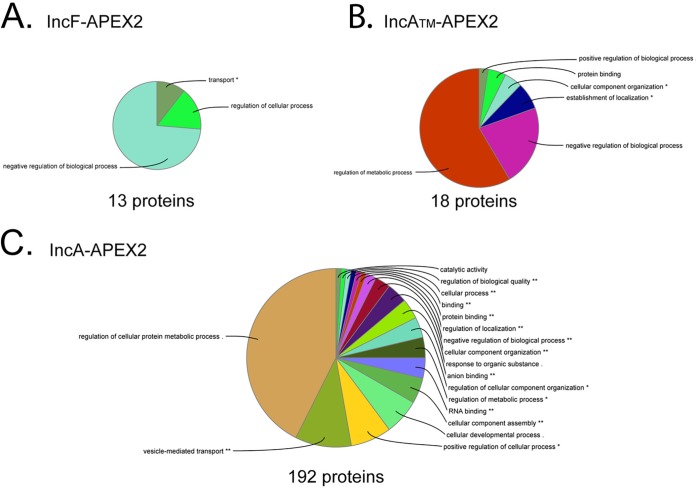
Visualization of global biological functions of AP-MS-identified and statistically significant eukaryotic proteins from Inc-APEX2 pulldowns. A ClueGO global network visualization of eukaryotic proteins identified by mass spectrometry (SAINT BFDR ≤ 0.05) from the C. trachomatis L2 IncF-APEX2 (A), IncA_TM_-APEX2 (B), and IncA-APEX2 (C) transformants is shown. See Fig. S2 and S3 in the supplemental material. The asterisks indicate significantly enriched GO terms: *, *P* < 0.05; **, *P* < 0.01.

When we applied SAINT to our Homo sapiens AP-MS data, 13 statistically significant eukaryotic proteins (BFDR ≤ 0.05) were identified using C. trachomatis L2 IncF-APEX2, 18 statistically significant proteins were identified using IncA_TM_-APEX2, and 192 statistically significant proteins were identified using IncA-APEX2 (Tables S2 and S3). To visualize common pathways for eukaryotic protein biological processes and molecular functions, the significant eukaryotic proteins (BFDR ≤ 0.05) from each SAINT-analyzed Inc-APEX2 data set were evaluated by use of the ClueGO plug-in (in Cytoscape software [48]) (Fig. 4). For IncF-APEX2 (Fig. 4A; Fig. S2A), the 13 significant eukaryotic proteins identified were associated with transport and the negative regulation of biological processes. For IncA_TM_-APEX2, the 18 statistically significant proteins were associated with the regulation of metabolic processes and biological processes (Fig. 4B; Fig. S2B). Finally, pathway analysis of the 192 significant eukaryotic proteins for IncA-APEX2 yielded globally enriched pathways, including the regulation of cellular protein metabolic processes, vesicle-mediated transport, actin cytoskeleton organization, the regulation of cellular component organization, and translation (Fig. 4C; Fig. S2C).

Individual data sets were also analyzed using the STRING tool (with Cytoscape software [48]) to visualize the protein binding partner network for statistically significant (BFDR ≤ 0.05) eukaryotic proteins within each IncF-APEX2 (Fig. S3A), IncA_TM_-APEX2 (Fig. S3B), and IncA-APEX2 (Fig. S3C) data set. Four statistically significant eukaryotic proteins were common to all Inc-APEX2 data sets: leucine-rich repeat flightless-interacting protein 1 (LRRF1 or LRRFIP1), microtubule-associated protein 1B (MAP1B), cystatin B (CYTB), and brain acid-soluble protein 1 (BASP1) (Table S2). Twelve proteins were shared between C. trachomatis L2 IncA-APEX2 and IncA_TM_-APEX2, including myosin phosphatase target subunit 1 (MYPT1 or PPP1R12A), transitional endoplasmic reticulum ATPase (TERA, VCP), microtubule-associated protein 4 (MAP4), multifunctional protein ADE2 (PUR6), sorting nexin 1 (SNX1), Src substrate cortactin (SRC8, CTTN), methylosome protein 50 (MEP50), sorting nexin 6 (SNX6), perilipin 3 (PLIN3), eukaryotic translation initiation factor 4B (IF4B), nucleoside diphosphate kinase A (NDKA), and nucleoside diphosphate kinase B (NDKB) (Table S2). In both the IncF-APEX2 and IncA-APEX2 data sets, four eukaryotic proteins were statistically significant: 14-3-3η (YWHAH), myristoylated alanine-rich C kinase substrate (MARCKS), 14-3-3β (YWHAB), and keratin type I cytoskeletal 20 (K1C20) (Table S2). These data included statistically significant eukaryotic proteins that have previously been shown to be recruited to the inclusion by Inc proteins. For example, in our IncA-APEX2 and IncF-APEX2 data sets, we identified 14-3-3β, which is known to bind IncG (9). In addition, the eukaryotic proteins SNX5 and SNX6, which bind IncE (24), and MYPT1, which binds CT228 (14, 15), were identified in both the IncA-APEX2 and IncA_TM_-APEX2 data sets (Table S2). Furthermore, the known chlamydial Inc binding partners for the eukaryotic proteins listed above (IncG, IncE, and CT228) were also identified in the AP-MS C. trachomatis L2 protein data sets analyzed (Table S1). We also identified eukaryotic proteins that are known to localize at the inclusion but for which an Inc binding partner has not been identified, including microtubule-associated protein 1B (MAP1B) (49) and Src-substrate cortactin (SRC8, CTTN) (50). A full summary of our data set compared to that of Aeberhard et al. (29) can be found in Table S4. Importantly, besides identifying eukaryotic proteins that are known to localize at the inclusion membrane, our Inc-APEX2 data identified several eukaryotic proteins that have not been previously examined for localization to the chlamydial inclusion.

### Colocalization of LRRF1 with the C. trachomatis L2 inclusion membrane.

One of the high-confidence AP-MS-identified eukaryotic proteins (significant in each Inc-APEX2 data set [BFDR = 0]), leucine-rich repeat in flightless-interacting protein 1 (LRRF1), has been reported to be involved in activating a type I interferon response (51–55), which plays a role in host cell clearance of intracellular bacteria during infection and the development of adaptive immunity (56). Also, a known LRRF1 binding partner called protein flightless 1 homolog (FLII) (BFDR = 0.02) (53, 57) was identified by SAINT analysis to be significant in the IncA-APEX2 data set (Table S2). FLII has been reported to associate with β-catenin to regulate its activity (58). In support of this finding, LRRF1 (24, 29, 31) and FLII (24, 29) were also identified in previous AP-MS experiments, and ectopically expressed FLII was shown to localize with the inclusion (29). Neither endogenous LRRF1 nor FLII has been examined for localization to the chlamydial inclusion.

**FIG 5:**
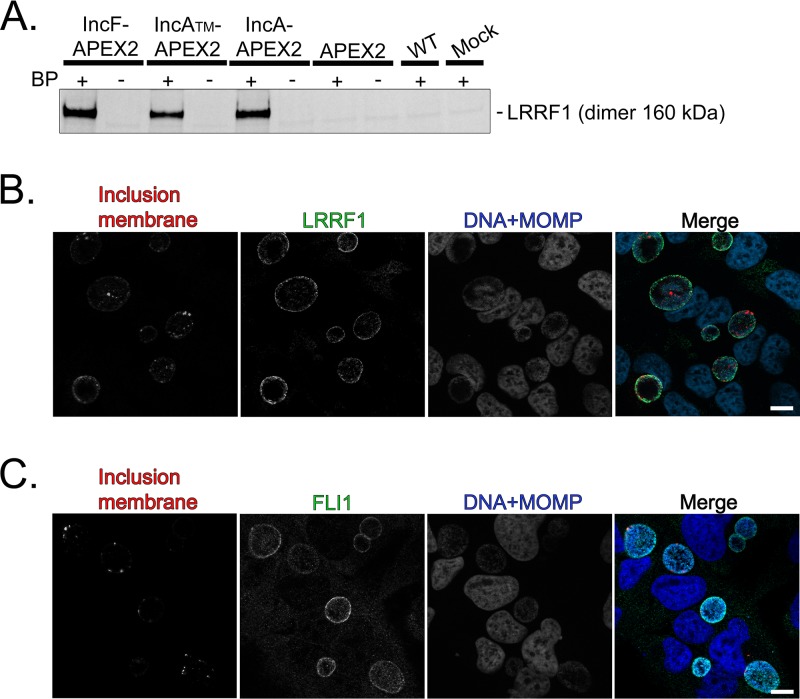
Confirmation of LRRF1 biotinylation by Inc-APEX2 proteins and localization of LRRF1 and FLII to the chlamydial inclusion. (A) Western blotting confirmation of LRRF1 in the eluates from streptavidin affinity-purified biotinylated lysate from the C. trachomatis L2 IncF-APEX2, IncA_TM_-APEX2, and IncA-APEX2 transformants at 24 hpi (BP, biotin-phenol). (B) Confirmation of LRRF1 colocalization with the inclusion of C. trachomatis L2 wild-type-infected HeLa cells. Cells were fixed at 24 hpi in 4% paraformaldehyde, permeabilized with 0.5% Triton X-100, and then stained for indirect immunofluorescence to visualize the inclusion membrane (CT223; red), LRRF1 (green), and DNA and chlamydiae (DRAQ5 and MOMP; blue). (C) Confirmation of FLII colocalization with the inclusion of C. trachomatis L2 wild-type-infected HeLa cells. Cells were fixed at 24 hpi in 4% paraformaldehyde, permeabilized with 0.5% Triton X-100, amd then stained for indirect immunofluorescence to visualize the inclusion membrane (CT223; red), FLII (green), and DNA and chlamydiae (DAPI and MOMP; blue). Coverslips were imaged using a Zeiss ApoTome.2 fluorescence microscope at ×100 magnification. Bars = 10 μm. See Fig. S4 in the supplemental material.

LRRF1 was first confirmed by Western blotting (dimer, 160 kDa) in the eluate from the streptavidin affinity-purified lysate from each of the C. trachomatis L2 Inc-APEX2-infected HeLa cells but not in the C. trachomatis L2 Inc-APEX2-infected samples that did not receive biotin-phenol, in the C. trachomatis L2 wild-type-infected sample, or in mock-infected negative-control samples (Fig. 5A). These data confirm the identification of LRRF1 by mass spectrometry. To assess if LRRF1 and FLII localized to the chlamydial inclusion, HeLa cells were infected with the C. trachomatis L2 wild type, fixed at 24 hpi, and stained for immunofluorescence. LRRF1 (Fig. 5B) and FLII (Fig. 5C) were observed to localize to the inclusion membrane at 24 hpi. Subsequently, we transfected HeLa cells with a vector encoding LRRF1 tagged with green fluorescent protein (GFP) (LRRF1-GFP) or FLII tagged with GFP (FLII-GFP), and then the cells were infected or not infected with the C. trachomatis L2 wild type. At 24 hpi, the cells were fixed and processed for immunofluorescence. In C. trachomatis L2-infected HeLa cells, LRRF1-GFP (Fig. S4A) and FLII-GFP (Fig. S4B) were each observed at the inclusion membrane. In support of this finding, ectopically expressed hemagglutinin epitope-tagged FLII was previously reported to localize to the C. trachomatis L2 inclusion (29). In mock-infected HeLa cells, both LRRF1-GFP and FLII-GFP appeared diffusely in the host cytosol (Fig. S4A and S4B, respectively). There was no significant difference in the inclusion area between C. trachomatis L2 wild-type-infected HeLa cells when LRRF1 was overexpressed (LRRF1-GFP-transfected cells) and nontransfected HeLa cells (Fig. S4C). When we knocked down LRRF1 expression in HeLa cells, we did not observe a biologically significant change in the production of infectious progeny (Fig. S4D; nontargeting small interfering RNA [siRNA] = 2.73 × 10^6^ inclusion-forming units [IFU]/ml; GAPDH [glyceraldehyde-3-phosphate dehydrogenase] siRNA = 4.46 × 10^6^ IFU/ml; single LRRF1 siRNA = 2.3 × 10^6^ IFU/ml; pooled LRRF1 siRNA = 4.09 × 10^6^ IFU/ml).

### LRRF1 colocalizes with the C. trachomatis inclusion from the mid to the late developmental cycle.

**FIG 6:**
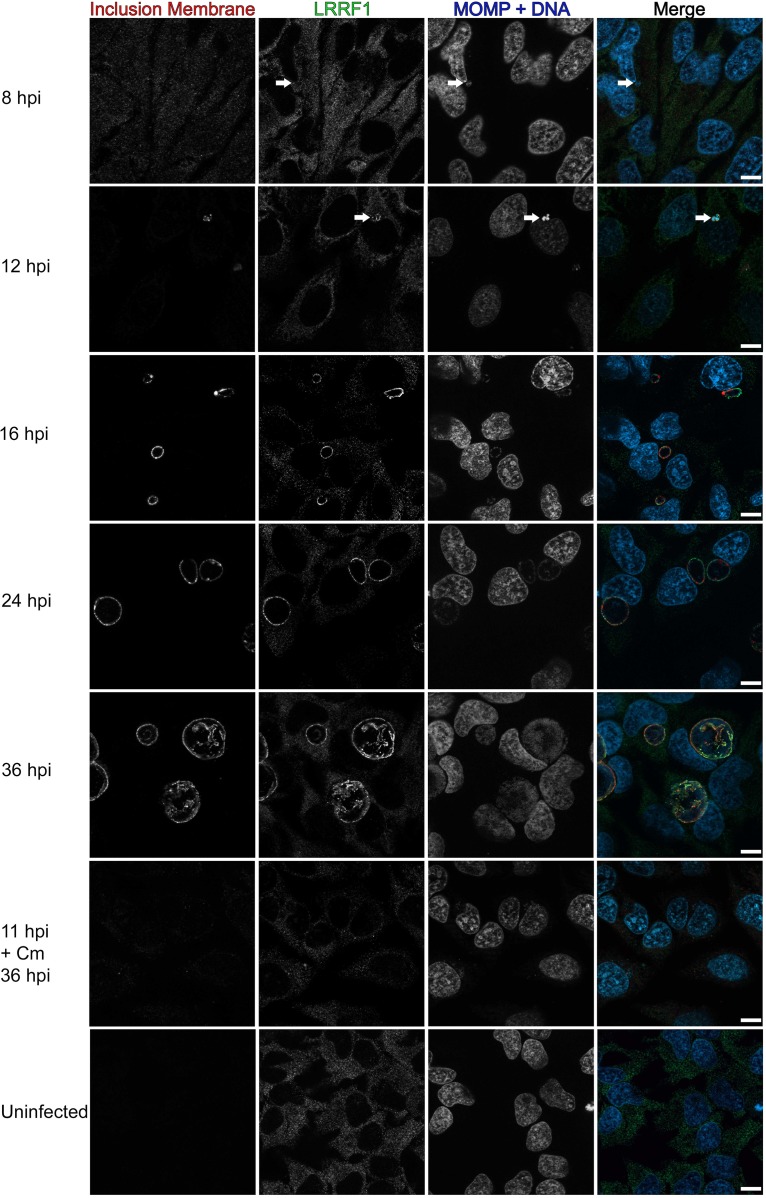
Recruitment of LRRF1 to the inclusion of C. trachomatis L2 during the developmental cycle and after chloramphenicol treatment. HeLa cells seeded on glass coverslips were infected with the C. trachomatis L2 wild type or were mock infected. The wells were methanol fixed at 8, 12, 16, 24, and 36 hpi. One sample was treated with 34 μg/ml chloramphenicol (Cm) at 11 hpi and fixed at 36 hpi. Fixed coverslips were stained for indirect immunofluorescence to visualize LRRF1 (green), the inclusion membrane (CT223; red), and DNA and chlamydiae (DAPI and MOMP; blue). Coverslips were imaged using a Zeiss ApoTome.2 fluorescence microscope at a ×100 magnification. Arrows indicate early inclusions at 8 hpi and LRRF1 colocalization with the inclusion at 12 hpi. Bars = 10 μm.

To determine if LRRF1 stably or transiently localized to the inclusion during the developmental cycle, we infected HeLa cells with the C. trachomatis L2 wild type, fixed the cells at intervals of between 8 hpi and 36 hpi, and then stained for immunofluorescence to observe LRRF1 localization. Using CT223 as an inclusion membrane marker, LRRF1 could be observed at the inclusion as early as 12 hpi (Fig. 6, arrows) and remained at the inclusion up to 36 hpi (Fig. 6). Chloramphenicol (Cm) was added at 8 hpi and 11 hpi (shown in Fig. 6) to inhibit bacterial translation, and this treatment at both time points abolished the localization of LRRF1 to the inclusion (Fig. 6, 11 hpi + Cm 36 hpi panels), suggesting that LRRF1 recruitment is dependent on active chlamydial protein expression. These data indicate that LRRF1 is stably localized to the inclusion membrane from mid to late time points in the C. trachomatis L2 developmental cycle and that a chlamydial protein may recruit LRRF1 to the inclusion.

### LRRF1 colocalization with the inclusion is conserved among several Chlamydia trachomatis serovars and *Chlamydia* species.

**FIG 7:**
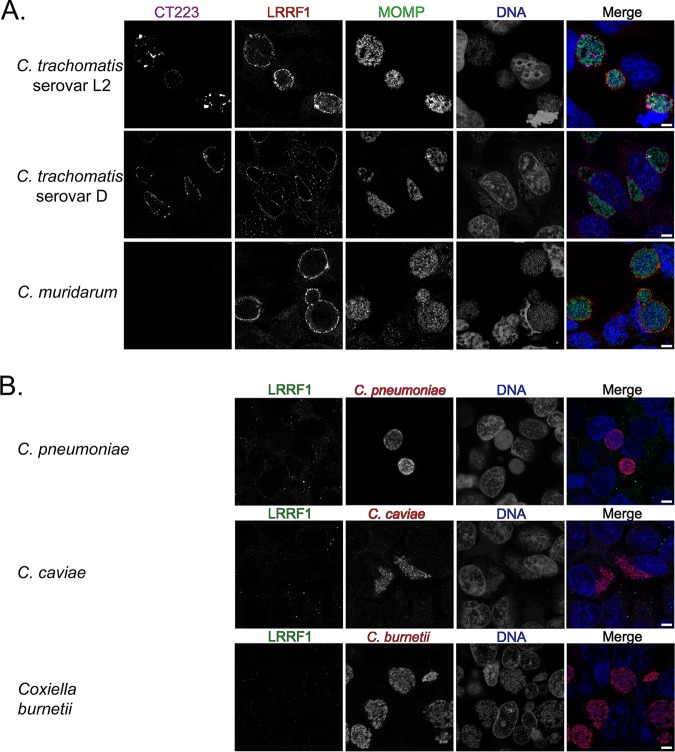
Examination of recruitment of LRRF1 to the inclusions of different chlamydial species and to the parasitophorous vacuole of the Coxiella burnetii Nine Mile phase II strain. (A) HeLa cells were infected with C. trachomatis serovar L2, C. trachomatis serovar D, or C. muridarum, fixed with methanol at 24 hpi, and stained for immunofluorescence to visualize the inclusion membrane (CT223; pink), LRRF1 (red), chlamydiae (MOMP; green), and DNA (DAPI; blue). CT223 is absent in C. muridarum. (B) HeLa cells were infected with C. pneumoniae and fixed in 4% paraformaldehyde at 96 hpi, with C. caviae and methanol fixed at 24 hpi, or with C. burnetii Nine Mile phase II and fixed with methanol at 3 days postinfection. Coverslips were stained for immunofluorescence to visualize LRRF1 (green), bacteria (red), and DNA (DRAQ5; blue) and imaged using a Zeiss LSM 800 confocal micrsocope at a ×63 magnification with a ×2 zoom. Bars = 5 μm.

LRRF1 contains a coiled-coil domain as well as a cytosolic nucleic acid binding domain (52, 54), indicating two possible modes of LRRF1 recruitment to the inclusion membrane. To test if LRRF1 recruitment was mediated by a bacterial protein or as part of an innate response to infection by an intracellular bacterium, HeLa cells were infected, fixed, and processed as indicated in Materials and Methods with various Chlamydia trachomatis serovars and *Chlamydia* species. An avirulent strain of Coxiella burnetii (Nine Mile phase II), which interacts with different eukaryotic pathways than *Chlamydia*, was also included (59). Our analysis of LRRF1 localization during infection of different *Chlamydia* species and C. trachomatis serovars revealed that LRRF1 colocalized with the inclusions of C. trachomatis serovar L2 (as observed above [Fig. 6]), C. trachomatis serovar D, and the closely related Chlamydia muridarum (Fig. 7A). LRRF1 did not colocalize with the inclusions of Chlamydia pneumoniae or Chlamydia caviae or with the *Coxiella*-containing vacuole of Coxiella burnetii (Fig. 7B). We conclude from these data that an Inc protein conserved among C. trachomatis serovar L2, C. trachomatis serovar D, and C. muridarum recruits LRRF1 to the inclusion membrane.

### BACTH assay to screen for LRRF1-Inc interacting partners.

To determine if the IncF and IncA used in the proximity labeling experiments can bind LRRF1, we used the bacterial adenylate cyclase two-hybrid (BACTH) system to screen for protein-protein interactions (23, 60, 61). Here, two plasmids encoding the proteins of interest genetically fused to the catalytic fragments (i.e., T25 and T18) of the Bordetella pertussis adenylate cyclase are cotransformed into Escherichia coli (Δ*cyaA*) (60, 62–64). An interaction between two proteins of interest brings the catalytic fragments in close proximity, restoring adenylate cyclase activity (60, 64, 65). Adenylate cyclase activity results in the production of cAMP and activates the expression of β-galactosidase via regulation of the chromosomally encoded *lac* operon in E. coli (60, 62–64). Positive interactions, indicated by the presence of blue colonies, are detected on minimal medium (supplemented with isopropyl β-d-1-thiogalactopyranoside [IPTG] and 5-bromo-4-chloro-3-indolyl-β-d-galactopyranoside [X-Gal]), and the interactions are quantified by a β-galactosidase assay (60, 62–64).

**FIG 8:**
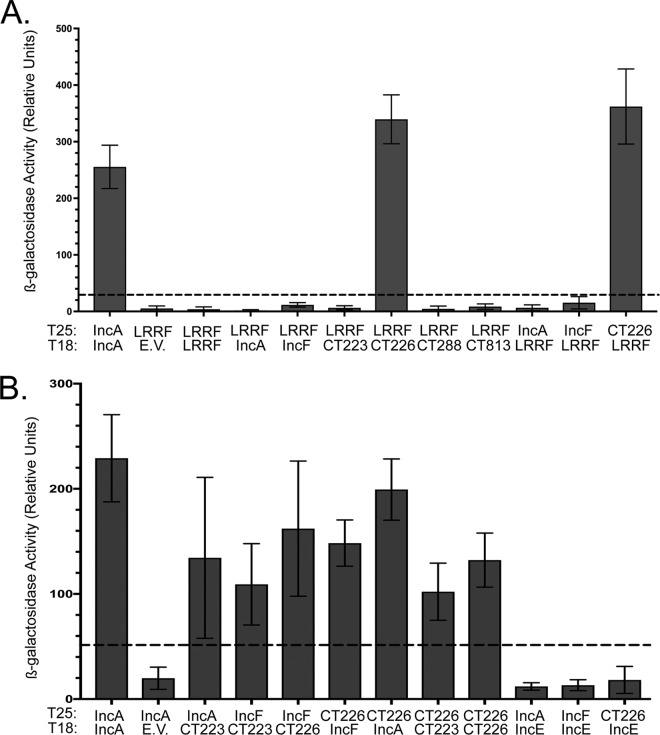
Bacterial adenylate cyclase two-hybrid (BACTH) assay to screen for LRRF1-Inc and Inc-Inc protein interactions. pST25 and pUT18 fused to chlamydial Incs or LRRF1 genes, as indicated, were cotransformed into E. coli DHT1 (Δ*cyaA*), plated on minimal medium containing IPTG and X-Gal, and grown for 3 to 5 days at 30°C. Colonies were picked for overnight culture, the interaction was quantified by a β-galactosidase assay, and the results are reported as relative units (RU). (A) Quantitative analysis of LRRF1-Inc interactions; (B) quantitative analysis of Inc-Inc interactions. Values greater than five times the value for the negative control (indicated by the dotted line) are considered a positive interaction. The data shown are the mean and standard deviation from three biological replicates, except for the IncE interactions, for which the data are representative of those for two biological replicates.

A targeted screen was performed using IncF, IncA, CT223, CT813, CT288, and CT226. These Incs either were detected in our proximity labeling experiments or are Incs that are conserved among C. trachomatis serovar L2, C. trachomatis serovar D, and C. muridarum (66). Of interest, LRRF1 contains a coiled-coil domain (54), which is a feature shared by several chlamydial Incs (36, 37, 67). Homotypic interactions have been previously described for IncA (23), which was used as a positive control. All interactions tested were quantified by the β-galactosidase assay (23, 68). No interaction was observed between LRRF1 and IncF, IncA, CT288, CT223, or CT813 (Fig. 8A). A positive interaction was detected between CT226 and LRRF1 (Fig. 8A), and this interaction is consistent with previously reported data (24). CT226, like LRRF1, contains a coiled-coil domain (67). The interaction between LRRF1 and CT226 appeared to be specific because no other Incs tested, even Incs with coiled-coil domains, yielded a positive interaction (Fig. 8A). In addition, the CT226 and LRRF1 interactions were positive in both BACTH plasmid conformations (e.g., T25-LRRF1 versus T18-CT226 and T25-CT226 versus T18-LRRF1).

We did not detect a positive interaction between LRRF1 and IncA or LRRF1 and IncF, our original Inc-APEX2 constructs. Instead, it is possible that LRRF1 may be proximal to but may not directly bind IncA and IncF at the inclusion membrane. To address this, we tested by BACTH the interactions of IncF and IncA with CT226, and both were found to interact with CT226 (Fig. 8B). CT226 also demonstrated homotypic interactions (Fig. 8B). Finally, we tested the ability of IncF and IncA to interact with CT223 (SAINT BFDR = 0), the statistically significant Inc identified from each IncF-APEX2, IncA_TM_-APEX2, and IncA-APEX2 data set. IncF and IncA each interacted with CT223 by BACTH (Fig. 8B). CT223 also interacted with CT226 by BACTH (Fig. 8B). In contrast, neither IncF nor IncA positively interacted with IncE (SAINT BFDR = 0.18 and 0.2, respectively), indicating the specificity of the BACTH interactions between the Incs tested (Fig. 8B) and indicating that the lack of interaction was not due to the lack of sufficient IncE expression during the BACTH assay (Fig. S5). These data support the likelihood that CT223 and CT226 are proximal to IncF and IncA in the inclusion membrane. The identification of the CT226-LRRF1 interaction by BACTH assay corresponds to both the immunofluorescence data (Fig. 7) and bioinformatic predictions because CT226 is conserved between C. trachomatis and C. muridarum but not between C. trachomatis and C. pneumoniae or C. caviae (66).

### Assessment of LRRF1 colocalization with chlamydial Incs in C. trachomatis L2-infected HeLa cells by superresolution microscopy.

To assess the spatial localization and proximity of LRRF1 with respect to IncA and IncF, we used structured illumination microscopy (SIM) with a superresolution microscope. We also examined the localization of CT226, which was identified by BACTH to be a potential partner interacting with LRRF1. Our IncA and IncF antibodies are both rabbit antibodies, as are the LRRF1 and FLII antibodies, precluding our ability to test endogenous IncF and IncA colocalization in C. trachomatis L2-infected eukaryotic cells. There are also no antibodies currently available to test if endogenous CT226 colocalizes with LRRF1 during C. trachomatis L2 infection. To assess the colocalization of LRRF1 with IncF and IncA, we used our C. trachomatis L2 IncF-APEX2, IncA_TM_-APEX2, and IncA-APEX2 transformants. In addition, we created C. trachomatis L2 transformed with a plasmid encoding CT226 fused to a FLAG tag (CT226_FLAG_) to test the localization between CT226 and endogenous LRRF1.

**FIG 9:**
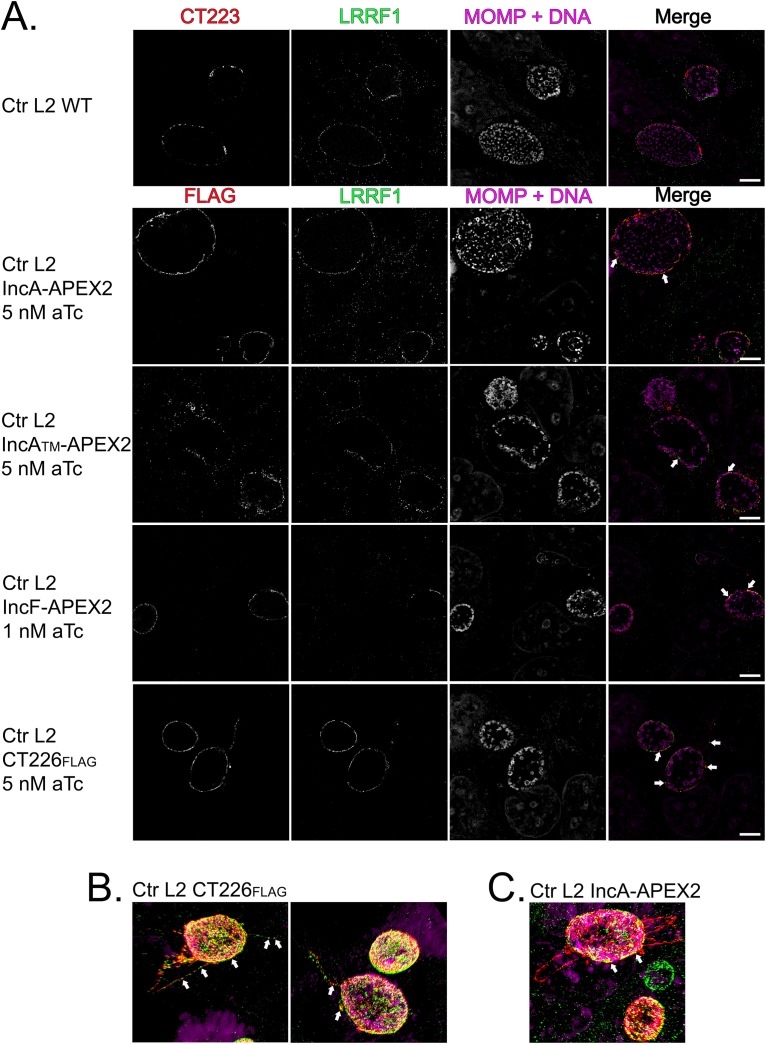
Assessment of LRRF1 colocalization with chlamydial Incs in C. trachomatis L2-infected HeLa cells using superresolution microscopy. (A) HeLa cells seeded on glass coverslips were infected with C. trachomatis L2 Inc-APEX2 transformants or CT226_FLAG_ transformants and induced for expression at 20 hpi (IncF-APEX2 was induced with 1 nM aTc; 5 nM aTc was used for all other transformants). At 24 hpi, the coverslips were fixed with ice-cold methanol and stained for immunofluorescence to visualize construct expression (FLAG) or CT223 (red), LRRF1 (green), and chlamydiae and DNA (DRAQ5 and MOMP; pink). Coverslips were imaged by structural illumination microscopy (SIM) with a Zeiss Elyra superresolution microscope at a ×63 magnification with a ×2 zoom. Bars = 5 μm. (B) SIM 3D snapshot of C. trachomatis L2 CT226_FLAG_-infected HeLa cells with CT226_FLAG_- and LRRF1-positive fibers. (C) SIM 3D snapshot of C. trachomatis L2 IncA-APEX2-infected HeLa cells with IncA fibers. Arrows indicate colocalization between the indicated expressed construct and LRRF1.

HeLa cells were infected with the C. trachomatis L2 wild type (i.e., a nontransformed strain) or the transformants and induced the strains (with 1 nM aTc for IncF-APEX2 and 5 nM for all other transformants) for construct expression at 20 hpi. At 24 hpi, the cells were fixed in ice-cold methanol and processed for immunofluorescence as described in Materials and Methods to detect the localization of IncF-APEX2, IncA_TM_-APEX2, IncA-APEX2, and CT226_FLAG_ with endogenous LRRF1. We assessed LRRF1 localization with endogenous CT223, the statistically significant chlamydial protein identified in each Inc-APEX2 data set (Fig. 9). Endogenous CT223 appeared in puncta, as previously observed (69, 70), and LRRF1 appeared to uniformly localize around the inclusion, consistent with our earlier localization data for LRRF1 (Fig. 5B). By SIM with a superresolution microscope, LRRF1 was found to colocalize with each Inc-APEX2 construct, supporting the identification of LRRF1 using each construct (Fig. 9). The expressed CT226_FLAG_ also colocalized with endogenous LRRF1 (Fig. 9). Interestingly, the expression of CT226_FLAG_ resulted in fibers staining for CT226 extending from the inclusion. These fibers were similar in appearance to IncA fibers (71). LRRF1 was also observed to colocalize with CT226 fibers (Fig. 9B; the arrows indicate the fibers). In contrast, LRRF1 did not colocalize with the fibers of the IncA-APEX2 constructs (Fig. 9C).

### Overexpression of CT226_FLAG_ from C. trachomatis L2 CT226_FLAG_ transformants results in increased LRRF1 and FLII at the inclusion membrane.

Next, we determined the effect of variable expression levels of CT226_FLAG_ from C. trachomatis L2 CT226_FLAG_ transformants on the recruitment of LRRF1 and FLII. HeLa cells were infected with C. trachomatis L2 CT226_FLAG_ and either not induced or induced for construct expression at 7 hpi using 5 nM aTc and 20 nM aTc. The coverslips were fixed at 24 hpi with a formaldehyde-glutaraldehyde solution, as indicated in Materials and Methods, and stained to visualize LRRF1, FLII, and CT226_FLAG_. All images were obtained using the same exposure (set to that for the samples induced with 20 nM aTc) on an LSM 800 confocal microscope at a ×63 magnification with a ×2 zoom. Increased amounts of LRRF1 and FLII were detected at the inclusion membrane upon increased expression of CT226_FLAG_ (Fig. S6). Note that LRRF1 was observed at the inclusion of the C. trachomatis L2 CT226_FLAG_ transformants not induced for the expression of CT226 using normal exposure levels (Fig. S7). These data support the recruitment of LRRF1 to the inclusion membrane by CT226 during C. trachomatis infection of HeLa cells.

### Coimmunoprecipitation of endogenous LRRF1 with C. trachomatis L2 CT226_FLAG_.

**FIG 10:**
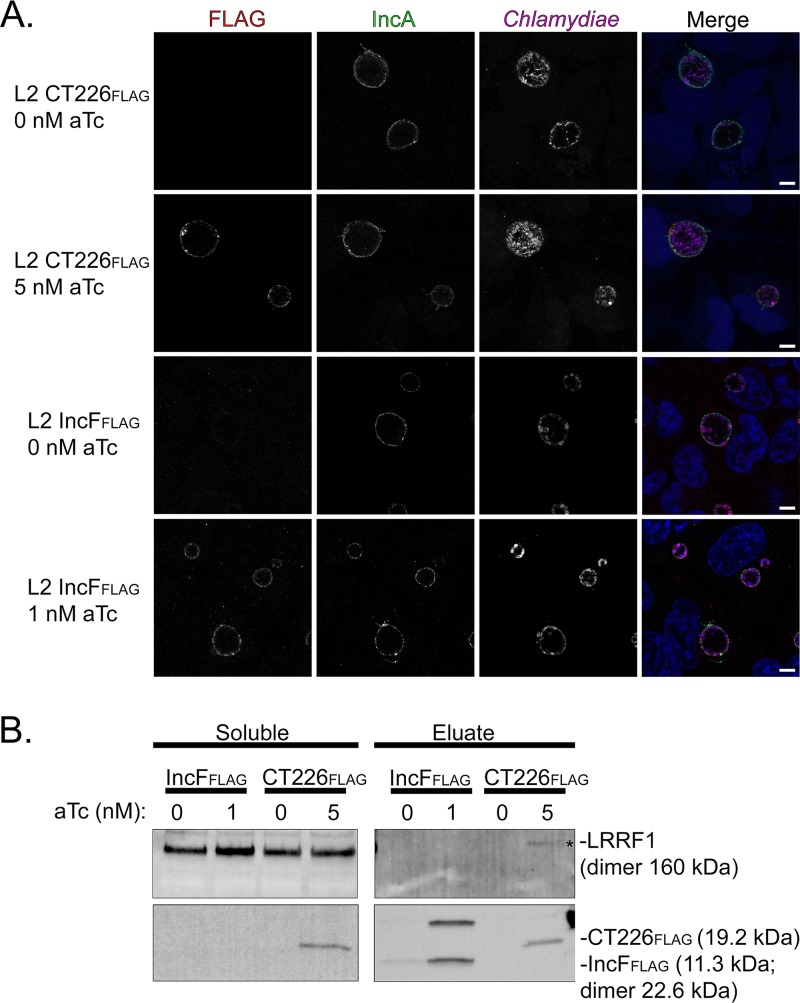
Coimmunoprecipitation of endogenous LRRF1 with C. trachomatis L2-expressed CT226_FLAG_. HeLa cells seeded in a 6-well plate with glass coverslips were infected with C. trachomatis L2 CT226_FLAG_ or IncF_FLAG_ and either not induced or induced for expression at 7 hpi with 5 nM aTc (CT226_FLAG_) or 1 nM aTc (IncF_FLAG_). (A) At 24 hpi, the coverslips were removed, fixed in 4% paraformaldehyde (L2 CT226_FLAG_) or methanol (L2 IncF_FLAG_), stained to visualize FLAG (red), the inclusion membrane marker (IncA; green), chlamydiae (MOMP; pink), and DNA (DAPI; blue), and imaged using a Zeiss LSM 800 confocal at a ×63 magnification with a ×2 zoom. Bars = 5 μm. (B) The remaining cells were collected, solubilized, normalized, and affinity purified using FLAG beads. The clarified lysates (soluble fraction) and eluate fractions were probed for construct expression (FLAG; CT226_FLAG_, 19.2 kDa; IncF_FLAG_, 11.3 kDa) and LRRF1 (dimer, 160 kDa). Three independent experiments were performed (see Fig. S6 in the supplemental material for additional replicates).

To test if LRRF1 directly bound to CT226, we performed coimmunoprecipitation assays with CT226_FLAG_ expressed from C. trachomatis-infected HeLa cells. HeLa cells were plated in 6-well plates containing glass coverslips to confirm construct expression and localization. The HeLa cells were infected with C. trachomatis L2 CT226_FLAG_ or FLAG-tagged IncF (IncF_FLAG_) transformants as a negative control. At 7 hpi, the constructs were either not induced or induced for expression using 5 nM aTc for C. trachomatis L2 CT226_FLAG_ and 1 nM aTc for IncF_FLAG_ (see reference 30 regarding the IncF induction conditions). At 24 hpi, the glass coverslips were removed, paraformaldehyde fixed, and processed for immunofluorescence, and then the cell lysates were collected and prepared for affinity purification using FLAG beads essentially as previously described (43). Both the clarified lysates (soluble fraction) and the eluates were blotted to detect each construct containing FLAG using an anti-FLAG antibody and each construct containing LRRF1 using an anti-LRRF1 antibody. Construct expression was observed by immunofluorescence for each C. trachomatis L2 CT226_FLAG_ or IncF_FLAG_ transformant, which were found to colocalize with the inclusion membrane marker IncA (Fig. 10A). The FLAG-tagged affinity-purified constructs were also detected by Western blotting (Fig. 10B; CT226_FLAG_, 19.2 kDa; IncF_FLAG_, 11.3 kDa [monomer] and 22.6 kDa [dimer]; Fig. S8). However, LRRF1 (dimer, 160 kDa) was detected only in the eluate fraction from the C. trachomatis L2 transformants induced for the expression of CT226_FLAG_ and not in those induced for the expression of IncF_FLAG_. These data further support our BACTH data, suggesting that LRRF1 can bind CT226_FLAG_ during C. trachomatis infection of eukaryotic cells. However, we cannot exclude the possibility that CT226_FLAG_ binds a third protein *in vivo* that recruits LRRF1, which results in coimmunoprecipitation with CT226_FLAG_.

## DISCUSSION

We previously reported the feasibility of using the ascorbate peroxidase (APEX2) proximity labeling system in C. trachomatis L2 to detect protein-protein interactions at the inclusion *in vivo* (30). This tool improves upon past techniques to understand protein-protein interactions by maintaining the spatial organization of Inc proteins in the inclusion membrane (30). Proteins proximal to and within the inclusion membrane can be biotinylated and identified by affinity purification (AP)-mass spectrometry (MS). Here, we used C. trachomatis L2 transformed with APEX2 fused to IncF and IncA, two Incs that, based on preliminary data, may represent distinct functional groups: Inc-Inc interactions to promote inclusion membrane organization and integrity or Inc-host protein interactions to facilitate chlamydia-host interactions and nutrient acquisition. As a control, we also prepared an IncA_TM_-APEX2 transformant that lacks the C-terminal SNARE-like domain of IncA and that more closely resembles IncF in size.

**FIG 11:**
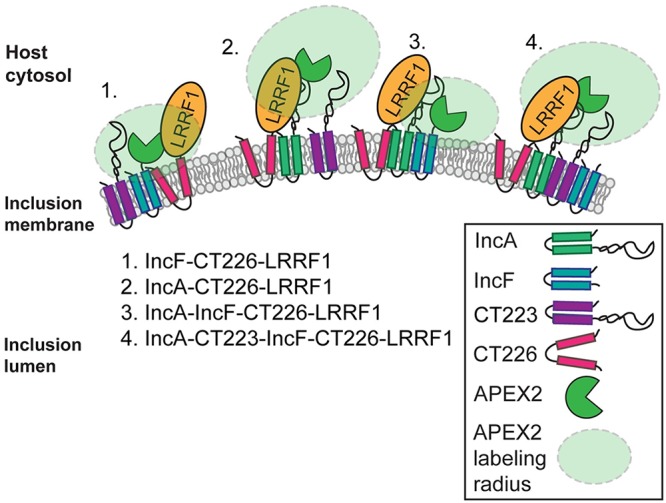
Model of Inc-Inc organization in the inclusion membrane and Inc-APEX2 proximity labeling. The proposed model of Inc organization is based on mass spectrometry-identified chlamydial Inc proteins, in which IncA-APEX2 and IncF-APEX2 proximity labeling constructs and bacterial adenylate cyclase two-hybrid (BACTH) assays were used to test protein-protein interactions. Based on these data, we propose four possible scenarios for the spatial organization of Incs and how these Incs were detected using the APEX2 proximity labeling system: scenario 1, IncF interacts with CT226, which binds LRRF1; scenario 2, IncA interacts with CT226, which binds LRRF1; scenario 3, IncA binds IncF and CT226, which binds LRRF1; and scenario 4, IncA, CT223, IncF, and CT226 (which binds LRRF1) all interact with each other. CT223 was statistically significant by SAINT analysis from the mass spectrometry data and was able to interact with IncF and IncA by BACTH.

As a field, we are at the early stages of understanding how the expression of various Inc constructs in the inclusion membrane can alter inclusion membrane organization and host protein recruitment. We focused on expressing our Inc-APEX2 constructs under conditions (e.g., timing of expression and amount of protein expressed) similar to those in which Incs are expressed endogenously. This is an important consideration, as overexpression of certain Incs can have deleterious effects on inclusion development and Inc localization (30) or can result in the recruitment of a greater abundance of eukaryotic proteins (see Fig. S6 in the supplemental material) that may or may not reflect *in vivo* conditions. This is in contrast to the findings of a recent study in which the authors overexpressed IncB-APEX2 (31), which did not result in the localization of IncB-APEX2 to microdomains within the inclusion membrane as endogenous IncB does (28, 50). The goal of this study may have been to identify all possible inclusion-proximal proteins, regardless of specificity. Here, we sought to understand the context for why a specific protein was prominent in our and others’ data sets, which ultimately revealed important information on how the inclusion membrane may be organized (Fig. 11).

To assign statistical significance and eliminate background contaminant proteins from the AP-MS data in an unbiased fashion, the proteins identified from H. sapiens and C. trachomatis L2 were analyzed by significance analysis of the interactome (SAINT) (35) (Table 1; Tables S1 to S3). This type of analysis is an improvement over the previously described statistical analyses used for similar data sets (31) because the *t* test and G test to determine whether to include a protein in the data set are not sufficient to estimate the false discovery rate (FDR). Newer methods, such as PepC, use a matrix of *t* test and G test confidence intervals to detect differentially expressed proteins (72). The SAINT method constructs separate distributions for true and false interactions to derive the probability of the observed bait-prey interaction. The probability model for the bait-prey interaction pair is used to estimate measurement errors in a transparent manner. SAINT generates a Bayesian false discovery rate (BFDR) calculation for each potential interaction detected in the data set. SAINT also normalizes the spectral counts on the basis of the protein length, which affects the potential availability of peptides that can be analyzed by MS/MS. These statistical tests are more rigorous than the independent *t* test and G test analyses (35, 73, 74, 94).

We applied the STRING interaction and ClueGo pathway analysis tools to our eukaryotic proteins whose interactions were identified to be significant (BFDR ≤ 0.05) by SAINT using the Inc-APEX2 constructs to detect globally enriched pathways. Consistent with our original hypothesis that IncA may preferentially interact with eukaryotic proteins compared to IncF, we detected a larger number of statistically significant eukaryotic proteins with our IncA-APEX2 construct (192 total) than with IncF-APEX2 (13 total), albeit with the caveats related to the labeling radius noted below. For IncA_TM_-APEX2, there was more than a log reduction in the number of proteins identified compared to the number identified with full-length IncA-APEX2, suggesting specificity for interactions at the C terminus of IncA. Given the presence of a SNARE-like domain in the C terminus of IncA, it is possible that the large number of proteins identified with IncA-APEX2 reflects its interactions with other SNARE proteins on vesicles carrying diverse cargo. For instance, vesicle-mediated transport (e.g., ANXA1, AP1M1, CAV1, GOLGA2, PDCD6, PDCD6IP, RAB34, RAB5B, SEC16A, SEC24C, SEC31A, SNX1, SNX2, SNX3, SNX5, SNX6, TFG, TSG101, USO1) has been described in the context of C. trachomatis L2 acquisition of specific lipids from Golgi apparatus-derived exocytic vesicles (7, 10, 11). Statistically significant hits involved in SNX retromer pathway disruption during chlamydial infection include SNX1, SNX2, SNX3, SNX5, SNX6, and SNX27 (24). Additional globally enriched biological processes and molecular functions involving cytoskeleton organization and translation align with previously published data. Hits involving cytoskeleton organization (e.g., ACTN1, ACTN4, CDC42, DPYSL3, DYNLL1, MARCKS, PLS3, RAC1, RHOA, SHTN1) correspond with those found in the literature, as the inclusion is surrounded by an F-actin cage (75–77). One of the four statistically significant eukaryotic proteins identified using each Inc-APEX2 construct, microtubule-associated protein 1B (MAP1B) (Table S2), would be expected because microtubules are known to surround the inclusion (49, 78, 79) and thus would be proximal to both IncF and IncA, which uniformly label the inclusion (21). This interpretation is consistent with the findings of another study using APEX2 (31).

Our AP-MS data also identified multiple statistically significant Inc proteins. Again, IncF-APEX2 labeled three different Incs, whereas only two were labeled for each of the IncA-APEX2 constructs (one of which was IncA itself). Consistent with our hypothesis, these data, taken together with the few eukaryotic proteins identified, suggest that IncF may preferentially interact with other Inc proteins. Importantly, IncA_TM_-APEX2 did not label more Incs than full-length IncA, even if we lowered the BFDR threshold. These data indicate specificity for the Incs identified with the IncF-APEX2 constuct. Although we identified chlamydial Incs with our Inc-APEX2 proximity labeling system, the majority of Incs detected were not statistically significant by SAINT (Table 1; Table S1). Some of this may reflect the residues that APEX2 covalently modifies during the biotinylation reaction (cysteine, histidine, tryptophan, and tyrosine residues) (32, 33, 42). The Incs that were significant (e.g., CT223 and IncA) have 11 to 20 cytosolically exposed target amino acids, whereas Incs that were not found to be statistically significant have less than 5 or 6 exposed target amino acids, in general. Therefore, proteins containing fewer APEX2-modifiable amino acids are not efficiently tagged with biotin and subsequently are not enriched as efficiently in the streptavidin affinity purification. There are also inherent difficulties in identifying hydrophobic proteins by mass spectrometry. To counter this difficulty, we included two enzymes to digest purified proteins into peptides, but these efforts, in combination with limited modifiable amino acids, may not have allowed for enough enrichment of APEX2-targeted Inc proteins. To compensate for this limitation, a lower BFDR significance threshold could be considered when analyzing chlamydial Inc proteins. For example, when we lowered the BFDR threshold to 0.2, we identified eight Incs from our AP-MS data, including IncA in the IncF-APEX2 data set that was detected by Western blotting (Fig. 3). Further experimentation and analysis of Inc-Inc APEX2 data are required to identify an appropriate cutoff.

Using chlamydial expressed Inc-APEX2 constructs, we identified several chlamydial Incs and their known interacting eukaryotic protein partners, including IncG and 14-3-3β (9), IncD and CERT (12, 13), CT228 and myosin phosphatase target protein subunit 1 (MYPT1) (14), and IncE and sorting nexin 5 (SNX5) and SNX6 (24) (Table 1; Tables S1 to S3). In addition, we identified eukaryotic proteins unique to each IncF-APEX2 and IncA-APEX2 data set that have not been demonstrated to localize to the chlamydial inclusion and that thus require further validation (Table S2). In support of our hypothesis that IncA might preferentially interact with eukaryotic proteins compared to IncF, more eukaryotic proteins were identified with IncA-APEX2 than with IncF-APEX2, as noted above (Table S2). We expanded on the current knowledge of proteins recruited to the inclusion membrane with the validation of two eukaryotic proteins not previously reported at the inclusion: LRRF1, which was statistically significant with each Inc-APEX2 data set, and its known binding partner, FLII, which was statistically significant with the IncA-APEX2 data set (Fig. 5 and 7; Table S2). We also identified a potential chlamydial protein-interacting partner for LRRF1 by BACTH: CT226 (Fig. 8A). These data are consistent with those described in a previous report of LRRF1 and CT226 potential interactions, identified by transfecting host cells with epitope-tagged CT226 followed by AP-MS (24).

We detected LRRF1 at the inclusion membrane, but LRRF1 knockdown does not negatively impact chlamydial progeny production in HeLa cells (Fig. S4C). One explanation for this may be that C. trachomatis already prevents the normal function of LRRF1 by sequestering it at the inclusion membrane. In this context, knockdown would not affect the production of infectious progeny. Alternatively, LRRF1 has been implicated in the production of a type I interferon response (52, 53), so a more relevant tissue culture model, such as human macrophages (80), which produce a robust interferon-mediated immune response, may be required. As no phenotype for LRRF1 knockdown was apparent, we chose to examine the nature of how LRRF1 was biotinylated by our constructs and ultimately identified in our data set, as it has also been found in previous AP-MS data sets (24, 29, 31). The BACTH assays (Fig. 8A) provide evidence for an interaction between LRRF1 and CT226. Further, the data obtained by SIM with a superresolution microscope indicated colocalization between LRRF1 and overexpressed CT226_FLAG_ (C. trachomatis L2 transformant) at the inclusion membrane as well as with CT226_FLAG_-positive fibers emanating from the inclusion (Fig. 9). Lastly, we identified LRRF1 in CT226_FLAG_ coimmunoprecipitations, indicating that these proteins are true binding partners during chlamydial infection. We conclude that LRRF1 was likely labeled by APEX2 because CT226 is adjacent to and likely interacts with IncA and IncF in the inclusion membrane, which would position it within the labeling radius of our APEX2 constructs (Fig. 11). We have attempted to make a CT226 knockout by allelic exchange but have been unsuccessful thus far. This may suggest that CT226 is essential or that the possible overexpression of CT225 and CT227 in the homology regions of the allelic exchange vector is deleterious. Alternatively, other genetic tools, such as TargeTron (81) or conditional knockdown by CRISPR interference (82), may successfully disrupt CT226 expression.

Our study has also revealed potential limitations of the APEX2 proximity labeling system to distinguish differences between Inc-protein binding partners and proteins that are in spatial proximity to the Incs at the inclusion membrane, as summarized in Fig. 11. For instance, the labeling radius of APEX2, at least in our hands, may be larger than that originally described (42). This would explain the identification of proteins proximal to IncF and IncA and not only specific protein binding partners at the inclusion (Fig. 11). For example, although we identified strong LRRF1 recruitment to the inclusion (Fig. 5), IncF and IncA, our bait proteins, were not identified to be interacting partners of LRRF1 by BACTH or using IncF_FLAG_ coimmunoprecipitations (Fig. 8A). In addition, the spatial organization of Incs in the inclusion membrane is not currently well understood, but IncA and IncF uniformly decorate the inclusion membrane, whereas CT223 localizes in discrete regions. *In vivo* two-hybrid experiments have shown that IncA and IncF interact (23), which might support the labeling of similar proximal proteins, and we identified IncA in IncF-APEX2-labeled eluates (Fig. 3). Another possible explanation for a larger labeling radius is related to the diffusion rate and half-life of biotin-phenol, which are approximately 1 ms (33). Diffusion of biotin-phenol would contribute to a greater labeling radius and identification of a larger pool of proteins. In support of the diffusion of biotin-phenoxyl radicals with our Inc-APEX2 constructs, we identified outer membrane complex B (OmcB) and major outer membrane protein (MOMP) in the AP-MS data (Table 1; Table S1). It is possible that during labeling with Inc-APEX2 transformants, biotin-phenoxyl diffuses across the inclusion membrane and labels the bacteria (intrainclusion) before the quenching step, and OmcB and MOMP are among the most abundant outer membrane chlamydial proteins. Shorter labeling times may decrease the labeling radius and increase the labeling specificity (83). Also, BACTH assays are a useful tool to determine protein-protein interactions *in vivo* using E. coli. In this study, using the BACTH assay to test chlamydial Inc-Inc protein interactions, we observed CT223-IncF and CT223-IncA interactions (Fig. 8B), which supports the identification of CT223 as a statistically significant Inc using each Inc-APEX2 construct (Table 1). However, we might miss some interactions with the BACTH system, if eukaryotic posttranslational modifications are required for the protein interactions to occur (e.g., phosphorylation [84]). Therefore, other validation methods, such as superresolution microscopy or the Duolink proximity ligation assay technology, provided that antibodies are available, are required. Alternatively, overexpression models could be used to detect interactions with epitope-tagged Incs (with the caveats noted above). Importantly, these data highlight the necessity of using adequate controls and statistical analyses to eliminate false positives and other proteins that may be transiently near the inclusion during the labeling period.

Our data highlight the utility of the ascorbate peroxidase proximity labeling system to detect novel protein interactions at the C. trachomatis inclusion membrane *in vivo*. This tool improves upon past techniques by maintaining the spatial organization of Incs in the inclusion membrane and biotinylating proximal proteins *in vivo*. Our goal was to determine if there is a preference for certain Incs in Inc-Inc interactions or Inc-eukaryotic protein interactions in the inclusion membrane using the AP-MS SAINT data as the foundation for further study. Determining the complex types of interactions that Incs orchestrate in the inclusion membrane will lead to a better understanding of how chlamydiae survive in their intracellular niche. Importantly, this technique is broadly applicable, when properly controlled, to other intracellular bacteria or parasites residing within a membrane-bound vacuole.

## MATERIALS AND METHODS

### Antibodies and reagents.

The primary antibodies used were mouse anti-FLAG (Sigma), rabbit anti-FLAG (Sigma), mouse antigiantin (Enzo), mouse anti-GAPDH (EMD Millipore), goat anti-MOMP (Meridian), rabbit anti-LRRF1 (Bethyl), rabbit anti-FLII (Thermo Fisher), rabbit anti-IncA (a kind gift from Ted Hackstadt, NIAID, Rocky Mountain Laboratories, Hamilton, MT), mouse anti-CT223 (a kind gift from R. Suchland, University of Washington, WA, and D. Rockey, Oregon State University, OR), mouse anti-C. trachomatis Hsp60 (a kind gift from Rick Morrison, University of Arkansas for Medical Sciences, Little Rock, AR), rabbit anti-C. burnetii (Elizabeth A. Rucks), and mouse anti-C. pneumoniae AR39 (a kind gift from H. Caldwell, NIAID, Bethesda, MD). The secondary antibodies used for immunofluorescence were donkey anti-647, -594, -488, and -405 or the streptavidin-488 conjugate. DRAQ5 and DAPI (4′,6-diamidino-2-phenylindole) were used to visualize DNA, as indicated below. Western blots were visualized using the appropriate secondary antibodies conjugated to IRDye 680LT or IRDye 800 CW (LiCor Biosciences, Lincoln, NE), and membranes were imaged using an Azure c600 system (Azure Biosystems, Dublin, CA) and processed using Adobe Photoshop Creative Cloud software (Adobe).

### Organisms and cell culture.

HeLa 229 cells (CCL-2.1; American Type Culture Collection [ATCC], Manassas, VA) were cultured at 37°C with 5% CO_2_ in biotin-free Dulbecco modified Eagle medium (DMEM; Gibco, Grand Island, NY) that was supplemented with 10% heat-inactivated fetal bovine serum (FBS; HyClone, Logan, UT), for routine tissue culture or with 1% FBS for experiments involving biotinylation, as previously described (30), and with 10 μg/ml gentamicin (Gibco, Grand Island, NY). HeLa cells were used to propagate Chlamydia trachomatis serovar L2 (lymphogranuloma venereum [LGV] strain 434) for purification using established protocols (85, 86). Chlamydial titers were determined using conventional protocols to establish multiplicities of infection (MOI), based on the number of inclusion-forming units (IFU), and were determined in HeLa cells as previously described (86, 87). McCoy cells (CRL-1696; ATCC, Manassas, VA) were cultured at 37°C with 5% CO_2_ in biotin-free DMEM (Gibco, Grand Island, NY) that was supplemented with 10% fetal bovine serum (FBS; HyClone, Logan, UT) and used for C. trachomatis L2 (LGV 434) transformation experiments. HeLa cells, McCoy cells, and density gradient-purified C. trachomatis strains were routinely tested for *Mycoplasma* spp. (Lookout Mycoplasma PCR detection kit; Sigma, St. Louis, MO). For some experiments, C. trachomatis serovar D (UW3-CX), C. muridarum, C. caviae, C. pneumoniae AR39, and avirulent Coxiella burnetii Nine Mile phase II (provided by Bob Heinzen, Rocky Mountain Laboratories, Hamilton, MT) were used (11).

### Creation of Inc fusion constructs for transformation into C. trachomatis L2.

All primers used in this study are listed in Table S5 in the supplemental material. All plasmids and E. coli strains used in the cloning projects are listed in Table S6. The Inc-APEX2 fusion constructs were made as previously described (30). pcDNA3 APEX2-NES was a gift from Alice Ting (plasmid number 49386; Addgene) (42). APEX2 contains a single N-terminal FLAG tag. For the construction of IncF_FLAG_, IncF with the C-terminal FLAG epitope was amplified from pTLR2 IncF-APEX2 (30) and cloned into pTLR2. CT226 was amplified from C. trachomatis L2 genomic DNA with primers containing a C-terminal FLAG tag and inserted into the mCherry site of pBOMB4-Tet (EagI/KpnI) (a gift from Ted Hackstadt, NIAID, Rocky Mountain Laboratories, Hamilton, MT) using an NEBuilder HiFi assembly cloning kit (New England Biolabs). The final constructs were transformed into a *dam*- and *dcm*-negative E. coli strain. All constructs were confirmed by sequencing (Eurofins MWG Operon, Huntsville, AL).

### Transformation of C. trachomatis L2.

Transformations were performed as described previously (88, 89). The Inc-APEX2 transformants (30) were plaque purified as described elsewhere (88, 90) and density gradient purified. Both pTLR2-IncF_FLAG_ and pBOMB4-Tet-CT226_FLAG_ were transformed as described above in the presence of 1 U/ml penicillin and 1 μg/ml cycloheximide.

### Electron microscopy determination of APEX2 activity and localization.

HeLa cells were seeded at 1.0 × 10^6^ cells/well in a 6-well plate containing 25 mm Thermanox cell culture-treated coverslips for electron microscopy (Nunc, Rochester, NY). To confirm construct expression using indirect immunofluorescence, glass coverslips were included in duplicate wells of a 24-well plate. The wells were infected with the C. trachomatis L2 wild type or the L2 IncF-APEX2, IncA_TM_-APEX2, IncA-APEX2, or APEX2-only transformants. C. trachomatis L2 IncF-APEX2 and IncA_TM_-APEX2 (MOI, 0.75) were infected by centrifugation in DMEM–10% FBS containing 2 U/ml penicillin and 1 μg/ml cycloheximide. Wells infected with C. trachomatis L2 IncA-APEX2 and APEX2 only (MOI, 0.75) received 1 U/ml penicillin and 1 μg/ml cycloheximide. The C. trachomatis L2 wild type was infected (MOI, 0.4) in DMEM–10% FBS containing 1 μg/ml cycloheximide. At 7 hpi, the transformants were induced with 0.3 nM aTc for L2 IncF-APEX2 and 5 nM for all other L2 transformants and the C. trachomatis L2 wild type. At 24 hpi, glass coverslips were fixed in 4% paraformaldehyde for 15 min at room temperature (RT), methanol permeabilized for 5 min, and then processed for immunofluorescence confirmation of construct expression as described above.

The wells intended for electron microscopy were prepared using a protocol adapted from that of Martell et al. (41). Briefly, the cells were washed with Dulbecco's phosphate-buffered saline (dPBS), fixing solution (2% glutaraldehyde and 2% paraformaldehyde in 0.1 M sodium cacodylate) was added to the wells, and the plate was incubated on ice for 1 h. All subsequent steps were performed on ice. The expressed APEX2 remains functional after fixation (using conditions with less than 4% formaldehyde). After 1 h, the wells were washed 5 times for 2 min each time with cold buffer A solution (0.1 M sodium cacodylate). To quench unreacted aldehyde groups, cold 20 mM glycine containing 2 mM CaCl_2_ in wash buffer A was incubated with the cells for 5 min. The wells were washed 5 times for 2 min each time with cold buffer A. To enhance the diffusion of the large molecule 3,3′-diaminobenzidine (DAB), the cells were pretreated with 0.5 mg/ml DAB in buffer A containing 2 mM CaCl_2_ for 30 min prior to the polymerization step. The pretreatment step allows the DAB to uniformly diffuse into the cells without being converted to the polymer (no H_2_O_2_ present). To catalyze the polymerization of DAB (regions proximal to APEX2), 0.5 mg/ml DAB and 3 mM H_2_O_2_ in buffer A containing 2 mM CaCl_2_ were added to the cells and the mixture was incubated for 30 min. Negative controls to determine background activity included the C. trachomatis L2 wild type with DAB treatment and C. trachomatis L2 IncA-APEX2 induced without DAB. Polymerized DAB is unable to diffuse from the subcellular compartment. Finally, the wells were washed 5 times for 2 min each time with cold buffer A and delivered to the University of Nebraska Medical Center Electron Microscopy Core to be processed. In brief, the samples were postfixed with 1% osmium tetroxide, stained with toluidine blue, dehydrated with a series of increasing ethanol concentrations, embedded, and sectioned. Sections were placed on 200-mesh uncoated copper grids (Ted Pella Inc.), stained with uranyl acetate and Reynold’s lead citrate, and examined using a Tecnai G2 Spirit (FEI) transmission electron microscope (TEM) operated at 80 kV. Representative electron micrographs are shown.

### FLAG affinity purification of APEX2 fusion constructs.

HeLa cells were seeded in a 6-well plate in DMEM–10% FBS and allowed to grow overnight. The cells were infected with C. trachomatis L2 IncF-APEX2, IncA_TM_-APEX2, IncA-APEX2, or APEX2 only (MOI, 0.75) in DMEM–10% FBS containing 1 μg/ml cycloheximide plus appropriate antibiotics (2 U/ml penicillin for C. trachomatis L2 IncF-APEX2 and IncA_TM_-APEX2, 1 U/ml penicillin for C. trachomatis L2 IncA-APEX2 and APEX2) and at 7 hpi were induced with 0.3 nM aTc (IncF-APEX2 only; see reference 30 regarding lower induction levels) and 5 nM aTc (all other C. trachomatis L2 transformants). Cell collection, the lysis procedure, and FLAG affinity purification were performed essentially as previously described (43). Briefly, at 24 hpi, the coverslips were removed from the respective wells and methanol fixed for 5 min at RT, and the remaining cells were scraped into dPBS and centrifuged at 900 × *g* for 10 min at 4°C. The pellets were resuspended in cell lysis buffer (50 mM Tris-HCl, pH 7.4, 150 mM NaCl, 0.5% sodium deoxycholate, 0.1% sodium dodecyl sulfate [SDS], 1% Triton X-100 [Sigma, St. Louis, MO], 1× Halt protease inhibitor cocktail [Thermo Scientific, Waltham, MA], universal nuclease [Pierce, Rockford, IL], 150 μM clasto-lactacystin β-lactone [Santa Cruz Biotechnology, Dallas, TX]). Equal volumes (EZQ protein quantification kit; Life Technologies, Carlsbad, CA) of clarified lysates were prepared for FLAG affinity purification with FLAG magnetic beads (Sigma, St. Louis, MO) and rotated for 2 h at 4°C. The affinity-purified proteins were eluted in 30 μl of lysis buffer (described above) containing FLAG peptide (200 μg/ml). The eluates from each sample were combined with an equal volume of 4× Laemmli sample buffer containing 5% β-mercaptoethanol and then loaded into a Criterion Midi 4 to 20% gradient SDS-PAGE gel (Bio-Rad, Hercules, CA). The gel was transferred to a polyvinylidene difluoride (PVDF) membrane (pore size, 0.45 μm; Thermo Scientific, Waltham, MA) and blotted using anti-FLAG antibody to detect construct expression. Clarified lysate (used as the input for the FLAG affinity purification) was electrophoresed and transferred to the PVDF membrane to blot for chlamydial Hsp60 as a loading control.

### Labeling with biotin-phenol and affinity purification of biotinylated proteins.

To identify proteins that were biotinylated using C. trachomatis L2 IncF-APEX2, IncA_TM_-APEX2, IncA-APEX2, and APEX2, HeLa cells in DMEM–1% FBS were seeded into one 6-well plate per condition (e.g., one plate for L2 IncF-APEX2 for the induced condition and one plate for L2 IncF-APEX2 for the uninduced condition). To monitor construct expression and biotinylation, coverslips were placed in two of the wells of the 6-well plate. The biotinylation assays were performed essentially as previously described (30, 32, 33). The cells were infected with C. trachomatis L2 IncF-APEX2, IncA_TM_-APEX2, IncA-APEX2, or APEX2 only (MOI, 0.75) with DMEM–10% FBS containing 1 μg/ml cycloheximide plus appropriate antibiotics (2 U/ml penicillin for C. trachomatis L2 IncF-APEX2 and IncA_TM_-APEX2, 1 U/ml penicillin for C. trachomatis L2 IncA-APEX2 and APEX2) and centrifuged at 400 × *g* at RT for 15 min. Penicillin and cycloheximide were present for all biotinylation experiments to preserve the integrity of our transformants and to minimize host cell background, respectively. The samples were induced for construct expression at 7 hpi with 0.3 nM aTc (IncF-APEX2 only; see reference 30 regarding lower induction levels) or 4 nM aTc (all other transformants). At 23.5 hpi, the cell monolayers were incubated with 1.5 mM biotinyl-tyramide (biotin-phenol; AdipoGen, San Diego, CA) for 30 min at 37°C in 5% CO_2_. At 24 hpi, the labeling process was catalyzed and quenched, and the lysate was collected as previously described (30). Normalized lysates (1 mg/ml) were added to equilibrated streptavidin magnetic beads (Pierce, Rockford, IL) and rotated for 90 min at RT. Proteins were eluted from the streptavidin magnetic beads by 4 min of incubation at 95°C in 2× Laemmli sample buffer containing 0.5 mM biotin. The eluates were loaded into Criterion Midi 4 to 20% gradient denaturing gels (Bio-Rad, Hercules, CA) in duplicate. The gel intended for Coomassie staining was resolved briefly (∼2 to 3 cm) and then stained (10% methanol, 5% acetic acid, Coomassie blue G). The duplicate gel, which was used for Western blotting confirmation of affinity purification, was resolved completely, and the gel was transferred to a PVDF membrane (pore size, 0.45 μm; Thermo Scientific, Waltham, MA) and blotted using the primary antibodies indicated above or a streptavidin-680 conjugate (Western blotting) and appropriate secondary antibodies conjugated to IRDye 680LT or IRDye CW or a streptavidin-IRDye 680LT conjugate (LiCor Biosciences, Lincoln, NE). The PVDF membranes were imaged using an Azure c600 system (Azure Biosystems, Dublin, CA) and processed using Adobe Photoshop Creative Cloud software (Adobe).

### Identification of biotinylated proteins using mass spectrometry.

Coomassie-stained gels were imaged, and then each lane was cut into six gel fractions to enhance the resolution of lower-abundance proteins. The UNMC Proteomics Core Facility performed in-gel digestion, preparation, and analysis of gel fractions. Protein fractions excised from the SDS-PAGE gel were destained, reduced with Tris-carboxyethyl phosphine, alkylated with iodoacetamide, and digested overnight with sequencing-grade trypsin (Promega, Madison, WI) and Asp-N (Promega, Madison, WI). Trypsin cleaves Lys and Arg residues, and Asp-N endoproteinase cleaves Asp and Cys residues. The peptides were eluted from the gel, concentrated to 20 μl by vacuum centrifugation, and analyzed using a high-resolution nano-liquid chromatography-tandem MS (MS/MS) system (Orbitrap Fusion Lumos Tribrid) coupled with a high-performance liquid chromatography system (UltiMate 3000; Thermo Scientific, Waltham, MA). Approximately 500 ng of peptides was run on the precolumn (75 μm by 2 cm; Acclaim PepMap 100; nanoViper; Thermo Scientific, Waltham, MA) and the analytical column (75 μm by 50 cm; Acclaim PepMap RSCL; nanoViper; Thermo Scientific, Waltham, MA). The samples were eluted using a 100-min linear gradient of acetonitrile (2.5 to 45%) in 0.1% formic acid.

All samples analyzed by MS/MS were analyzed using the Mascot (version 2.6) server (Matrix Sciences, London, UK). The Mascot server was set up to search the Swiss-Prot database (selected for Homo sapiens 2017_02 [20,286 entries] and C. trachomatis strain 434/Bu entries), assuming that the digestion enzymes were trypsin and Asp-N. The parameters on the Mascot server were set as follows: enzyme, trypsin and Asp-N for biological replicates (*n* = 5); maximum missed cleavages, 2; peptide charges, 1+, 2+, and 3+; peptide tolerance, ±0.8 Da; fixed modifications, carbamidomethyl (C); variable modifications, oxidation (M) and biotin-phenol (C, Y, W, H); MS/MS tolerance, ±0.6 Da; and instrument, ESI-TRAP. Proteins identified by the Mascot search were uploaded into Scaffold software for visualization of the identified proteins (Proteome Software, Inc., Portland, OR).

### Statistical analysis of mass spectrometry samples using SAINT.

Significance analysis of the interactome (SAINT) was performed to assign statistical significance (Bayesian false discovery rate [BFDR]) to our mass spectrometry data (35). SAINT calculates the probability that a protein identified in the test sample is a true interacting protein, based on the average number of hits in the test samples compared to the control samples in an unbiased fashion. Scaffold files containing each replicate (*n* = 5) were set to a 95% protein threshold, 1 peptide minimum, and a 95% peptide threshold, and the sample report was exported to an Excel file. The sample report file was used to make three files required for SAINT analysis: the bait, prey, and interaction files (Table S1 and S3). The bait file corresponds to the sample condition (e.g., IncF-APEX2, replicate 1, test condition) and assigns samples as either a test or a control. For our data set, the Inc-APEX2 proteins biotinylated via IncF-APEX2, IncA_TM_-APEX2, and IncA-APEX2 were the test (T) samples, and the controls (C) were assigned to APEX2-, the L2 wild-type-, and mock-infected HeLa cells. The prey file is the list of all proteins from the Scaffold sample report file with their gene names and amino acid length (obtained from UniProt). The last file required for SAINT is the interaction file, which assigns the biological replicate number and spectral counts for each protein identified in the test subjects and the control samples. These files are input to calculate the Bayesian false discovery rate (BFDR) and were used to prioritize which proteins were statistically significant (35). We then input high-confidence data (BFDR ≤ 0.05) into the pipeline to visualize interaction networks, created using the STRING database (interaction confidence, 0.7; STRING). The defined STRING networks were exported and analyzed using Cytoscape (version 3.7.1) software (48) with the ClueGo plug-in to determine the globally enriched biological processes and molecular functions within each data set.

### Transfection of LRRF1-GFP and FLII-GFP.

The LRRF1 detected by mass spectrometry corresponded to LRRF1 variant 3. To assess LRRF1 and FLII localization during C. trachomatis L2 infection, we obtained pCMV6-AC-LRRF1-GFP (LRRF1 variant 3; catalog number RG226542; Origene, Rockville, MD) and pCMV6-AC-FLII-GFP (catalog number RG206863; Origene, Rockville, MD), respectively. For DNA transfections, 8 × 10^4^ HeLa cells per well were seeded onto 12-mm glass coverslips in a 24-well plate. Approximately 24 h later, fresh DMEM–10% FBS (antibiotic free) was added to the cells. The transfection efficiency was first optimized using various nanogram amounts of plasmid DNA (pDNA) and volumes of the jetPRIME transfection reagent (Polyplus, Illkirch, France). Optimal efficiency was determined with 100 ng of pCMV-LRRF1-GFP or 500 ng of pCMV6-AC-FLII-GFP added to 50 μl of jetPRIME buffer and 1.0 μl of transfection reagent (Polyplus, Illkirch, France). Samples were vortexed for 10 s, centrifuged briefly, and incubated at RT for 10 min. The plasmid-transfection reagent mixture was added dropwise to individual wells. After 4 h posttransfection, the medium was changed, and 2 h later (at 6 h posttransfection), HeLa cells were infected with the C. trachomatis L2 wild type (MOI, 0.8) by centrifugation at 400 × *g* for 15 min at RT. At 24 hpi, the cells were fixed in 4% paraformaldehyde, permeabilized with 0.5% Triton X-100 for 5 min at RT, and stained for immunofluorescence to visualize the inclusion membrane (anti-CT223), LRRF1-GFP, FLII-GFP, and DNA (DAPI). The coverslips were imaged using a Zeiss ApoTome.2 fluorescence microscope at a ×100 magnification. Inclusion area measurements were also taken for LRRF1-GFP-transfected HeLa cells infected with the C. trachomatis L2 wild type and compared to the inclusion area for nontransfected cells. The inclusion area is reported as the total for LRRF1-GFP-transfected HeLa cells (the inclusions from both high- and low-LRRF1-GFP-expressing cells) and individually for LRRF1-GFP-transfected HeLa cells with high and low levels of expression only (see the inset in Fig. S4C in the supplemental material). A minimum of 100 inclusions were measured for nontransfected samples, and a minimum of 100 inclusions were measured for HeLa cells with high levels of LRRF1-GFP expression and low levels of LRRF1-GFP expression (see the inset in Fig. S4C). Two independent experiments were performed. The inclusion area was graphed in GraphPad Prism (version 7) software, and a one-way analysis of variance with Tukey’s multiple-comparison *post hoc* test was performed to determine statistical significance.

### siRNA knockdown of LRRF1 to determine the effect on infectious progeny production.

siRNA knockdown experiments were performed following the manufacturer’s protocol (Polyplus, Illkirch, France). Nontargeting siRNA (catalog number SR30004; Origene, Rockville, MD), GAPDH siRNA (catalog number 4390849; Ambion), and pooled LRRF1 siRNA (catalog numbers 43450, s229968, and s17599; Ambion Life Technologies) were used in knockdown experiments. siRNA experiments were set up in quadruplicate to confirm the LRRF1 knockdown efficiency by Western blotting (one well), to detect LRRF1 (unpublished data) or FLII localization by immunofluorescence (one well), and to quantify infectious progeny (two wells). Briefly, 20 nM the nontargeting siRNA, GAPDH siRNA, single LRRF1 siRNA, or pooled LRRF1 siRNA was added to serum-free Opti-MEM medium (100 μl/well), and 2 μl of INTERFERin reagent (Polyplus, Illkirch, France) was added to each well. The wells were incubated for 15 min at RT with gentle rocking. Then, 2.5 × 10^4^ HeLa cells were added to each well on top of the siRNA-transfection reagent mixture, and the plate was incubated at 37°C in 5% CO_2_. The medium was replaced with fresh DMEM–10% FBS after 24 h. At 48 h post-siRNA transfection, HeLa cells were infected with the C. trachomatis L2 wild type (MOI, 0.8) by centrifugation at 400 × *g* for 15 min at RT.

At 30 hpi, to confirm the knockdown efficiency, C. trachomatis L2-infected HeLa cells were trypsinized, centrifuged, and resuspended in 2× Laemmli sample buffer. The lysate was loaded, electrophoresed, transferred to a PVDF membrane, and then blotted to detect the presence of LRRF1 and GAPDH. To measure the infectious progeny, experiments were performed essentially as previously described (91, 92). Briefly, at 30 hpi the cells from duplicate wells for each sample were scraped from the wells and lysed, and then the lysates were centrifuged at 17,000 × *g* for 30 min at 4°C. The pellet was resuspended in sucrose phosphate (2SP) buffer, serially diluted, and used to infect in duplicate a fresh monolayer of HeLa cells by centrifugation at 400 × *g* for 15 min at RT. The cells were incubated at 37°C in 5% CO_2_ for 15 min, and then the 2SP buffer was replaced with DMEM–10% FBS containing 1 μg/ml cycloheximide. To quantify the infectious progeny, at 24 to 30 h post-secondary infection, the cells were fixed in methanol for 5 min at RT and processed for indirect immunofluorescence to visualize the inclusion using anti-MOMP antibodies (Meridian Biosciences, Memphis, TN). The average number of inclusion-forming units per milliliter from three biological replicates is reported.

### Validation of LRRF1 at the chlamydial inclusion and time course experiments.

HeLa cells infected with C. trachomatis L2 (MOI, 0.75) in DMEM–10% FBS without antibiotics were fixed at 24 hpi in 4% paraformaldehyde, permeabilized with 0.5% Triton X-100 for 5 min at RT, and stained for immunofluorescence to visualize the inclusion membrane (anti-CT223), LRRF1, and DNA (DAPI). The coverslips were imaged using a Zeiss ApoTome.2 fluorescence microscope at a magnification of ×100.

For the time course experiments, HeLa cells infected with C. trachomatis L2 (MOI, 0.75) or mock infected in DMEM–10% FBS without antibiotics were fixed at 8, 12, 16, 24, and 36 hpi in methanol for 5 min at RT. One sample was treated with 34 μg/ml chloramphenicol at 8 hpi and 11 hpi (shown in Fig. 6) and then methanol fixed at 36 hpi. Fixed coverslips were stained for immunofluorescence to visualize the inclusion membrane (anti-CT223), LRRF1, chlamydiae (MOMP), and DNA (DAPI) and imaged using a Zeiss ApoTome.2 fluorescence microscope at a magnification of ×100.

### Assessing LRRF1 localization during infection of C. trachomatis serovars, *Chlamydia* species, and Coxiella burnetii.

HeLa cells infected with C. trachomatis L2 (MOI, 0.75), C. trachomatis serovar D (MOI, 1), C. muridarum (MOI, 0.25), C. caviae (MOI, 0.25), C. pneumoniae (MOI, 1), and avirulent Coxiella burnetii Nine Mile phase II were used. DMEM–10% FBS medium did not contain antibiotics (penicillin or cycloheximide) for these experiments. C. trachomatis serovar D was pretreated with DEAE-dextran prior to infection. All *Chlamydia*-infected HeLa cells were centrifuged at 400 × *g* for 15 min at RT. C. burnetii Nine Mile phase II-infected HeLa cells were centrifuged for 1 h at 2,000 rpm at RT. At 24 hpi, C. trachomatis L2-, C. trachomatis serovar D-, C. muridarum-, and C. caviae-infected HeLa cells were methanol fixed and stained for immunofluorescence. At 96 hpi, C. pneumoniae-infected HeLa cells were fixed in 4% paraformaldehyde, permeabilized with 0.5% Triton X-100, and stained for immunofluorescence. At 3 days postinfection, C. burnetii Nine Mile phase II-infected HeLa cells were methanol fixed for 5 min at RT and stained. Coverslips were stained using the organism-specific and LRRF1 antibodies listed above in “Antibodies and reagents” to examine LRRF1 localization and DRAQ5 or DAPI to visualize the DNA.

### Bacterial adenylate cyclase two-hybrid (BACTH) assays.

To screen for LRRF1-interacting partners, the LRRF1 gene was amplified from the pCMV-LRRF1-GFP vector (Origene, Rockville, MD), and Inc protein genes were amplified from C. trachomatis L2 genomic DNA using primers with overlapping sequences for each pST25 and pUT18C vector (Tables S5 and S6). The genes for LRRF1, CT288, CT226, CT223, IncA, IncF, and IncE were amplified using the primers listed in Table S6, cloned into either pST25 or pUT18C using a NEBuilder HiFi assembly cloning kit (NEBuilder; New England Biolabs), and transformed into E. coli DH5α *lacI*^q^. Individual clones were cultured overnight, pDNA was isolated (Qiagen, Germantown, MD) and verified by restriction digestion, and the final clones were verified by DNA sequencing. pUT18C-IncF (serovar D; Gateway) was made as previously described (23). To screen for interactions, assays were performed as described previously (23, 61, 64, 68, 93). Briefly, plasmids were cotransformed into E. coli DHT1 (Δ*cyaA*) (Table S6), and prior to plating, the E. coli cells were pelleted, washed, and resuspended in 1× M63 minimal medium. The resuspended E. coli DHT1 cells were then plated on 1× M63 minimal medium plates containing 0.2% maltose, isopropyl β-d-1-thiogalactopyranoside (IPTG; 0.5 mM), 5-bromo-4-chloro-3-indolyl-β-d-galactopyranoside (X-Gal; 0.04 mg/ml), Casamino Acids (0.04%), spectinomycin (25 μg/ml), and ampicillin (50 μg/ml) and incubated at 30°C for 3 to 5 days. To quantify interactions by a β-galactosidase assay, eight colonies (or streaks from negative plates) were set up for overnight culture in minimal medium (1× M63 minimal medium containing 0.2% maltose, 0.5 mM IPTG, 0.04 mg/ml X-Gal, 0.01% Casamino Acids, spectinomycin [25 μg/ml], and ampicillin [50 μg/ml]). After 20 to 24 h, the cultures were diluted, and the optical density at 600 nm (OD_600_) was measured. A duplicate set of samples was permeabilized with SDS (0.05%) and chloroform. After permeabilization, the supernatant was transferred to an optical plate containing 0.4% *o*-nitrophenyl-β-d-galactopyranoside in PM2 buffer (70 mM Na_2_HPO_4_·12 H_2_O, 30 mM NaH_2_PO_4_·H_2_O, 1 mM MgSO_4_, 0.2 mM MnSO_4_; pH 7.0 [23]) with 100 mM 2-mercaptoethanol. After 20 min, the enzymatic reaction was stopped with 1 M Na_2_CO_3_ stop solution and the absorbance at 405 nm was measured. The OD_405_ was normalized to bacterial growth (OD_600_) and reported as relative units (RU). A positive interaction was defined as a value greater than five times that for the negative control (63). The results of three independent experiments were analyzed for each interaction, were graphed using GraphPad Prism (version 7) software, and are reported as the mean with the standard deviation.

### Superresolution microscopy to assess LRRF localization with Incs.

HeLa cells seeded on glass coverslips were infected with the C. trachomatis L2 wild type or C. trachomatis L2 transformants IncF-APEX2, IncA_TM_-APEX2, IncA-APEX2, and CT226_FLAG_ and induced for expression at 20 hpi (induction was with 5 nM aTc for all transformants except IncF-APEX2, which was induced with 1 nM aTc). At 24 hpi, the coverslips were rinsed once with dPBS and then fixed with ice-cold methanol and stained for immunofluorescence to visualize construct expression (FLAG) or CT223 (red), LRRF1 (green), chlamydiae, and DNA (blue). The coverslips were imaged using structured illumination microscopy (SIM) with a Zeiss Elyra PS.1 superresolution microscope. Using Zen (blue edition; Zeiss) software, three-dimensional (3D) snapshots from C. trachomatis L2 CT226_FLAG_-infected HeLa cells and C. trachomatis L2 IncA-APEX2-infected HeLa cells with IncA fibers were generated and exported for visualization.

### Overexpression of CT226_FLAG_ from C. trachomatis L2 CT226_FLAG_ transformants results in increased LRRF1 and FLII levels at the inclusion membrane.

HeLa cells seeded on glass coverslips were infected with the C. trachomatis L2 CT226_FLAG_ transformants and either not induced or induced for expression at 7 hpi using 5 nM or 20 nM aTc. At 24 hpi, coverslips were fixed with 3% formaldehyde and 0.022% glutaraldehyde in dPBS, permeabilized with methanol, and stained for immunofluorescence to visualize construct expression (FLAG; red), chlamydiae (MOMP; gray), DNA (DAPI; blue), and either LRRF1 (green) or FLII (green). Coverslips were imaged using a Zeiss LSM 800 confocal microscope at a ×63 magnification with a ×2 zoom. Images were captured using the same exposure time (set to that for the samples induced with 20 nM aTc) for uninduced and 5 nM aTc-induced samples.

To examine LRRF1 recruitment using a normal exposure time, HeLa cells seeded on glass coverslips were infected with C. trachomatis L2 CT226_FLAG_ transformants and either not induced or induced for expression at 7 hpi using 5 nM aTc. At 24 hpi, coverslips were fixed with 4% paraformaldehyde, permeabilized with 0.5% Triton X-100, and stained for immunofluorescence to visualize construct expression (FLAG; red), LRRF1 (green), GFP-expressing chlamydiae (pseudoblue), and DNA (DAPI; blue). Coverslips were imaged using a Zeiss ApoTome.2 fluorescence microscope at a ×100 magnification.

### Coimmunoprecipitation of CT226_FLAG_ with endogenous LRRF1.

HeLa cells were seeded in a 6-well plate in DMEM–10% FBS and allowed to grow overnight. A coverslip was placed in two wells of each 6-well plate to monitor construct expression and localization by indirect immunofluorescence for each experiment. The cells were infected with C. trachomatis L2 CT226_FLAG_ and IncF_FLAG_ (MOI, 0.8) in DMEM–10% FBS containing 1 U/ml penicillin (but not cycloheximide) and not induced or induced with 5 nM (CT226_FLAG_) or 1 nM (IncF_FLAG_) aTc at 7 hpi. At 24 hpi, the coverslips were removed, fixed in 4% paraformaldehyde, permeabilized with Triton X-100 (0.5%), and stained for immunofluorescence to detect construct expression (FLAG), the inclusion membrane (IncA), DNA, and chlamydiae. The cells were lysed and affinity purified using FLAG magnetic beads as described above and previously (43). The eluates were mixed with an equal volume of 4× Laemmli sample buffer containing 5% β-mercaptoethanol and then loaded into a Criterion Midi 4 to 20% gradient SDS-PAGE gel (Bio-Rad, Hercules, CA). The gel was transferred to a PVDF membrane (pore size, 0.45 μm; Thermo Scientific, Waltham, MA) and blotted using anti-FLAG antibody to detect construct expression and anti-LRRF1 antibody. Three biological replicates were analyzed by coimmunoprecipitation.

## Supplementary Material

Supplemental file 1

Supplemental file 2

Supplemental file 3

Supplemental file 4

Supplemental file 5

Supplemental file 6

Supplemental file 7
