# Supplementary material for: Proximity Labeling To Map Host-Pathogen Interactions at the Membrane of a Bacterium-Containing Vacuole in Chlamydia trachomatis-Infected Human Cells
Source: Infect Immun. 2019 Oct 18;87(11):e00537-19. doi: 10.1128/IAI.00537-19 (PMC6803327; doi:10.1128/IAI.00537-19)
Supplement: Supplemental file 1 [file IAI.00537-19-s0001.pdf]

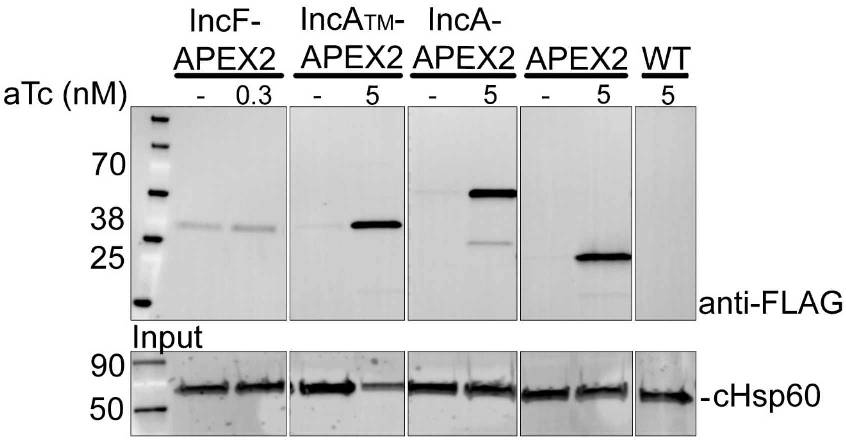

Fig S1. Western blot detection of the expressed APEX2 constructs.

HeLa cells infected with *C. trachomatis* L2 transformants or wild-type (WT) were induced with anhydrotetracycline (aTc) at 7 hpi (0.3 nM for IncF-APEX2; all other samples 5 nM), or not induced as indicated. Lysates were collected at 24 hpi, solubilized, and affinity purified using FLAG beads. The eluates were separated by electrophoresis, transferred to PVDF membrane, and blotted for APEX2 containing constructs using anti-FLAG antibody. The total lysate (input) was probed with anti- *C. trachomatis* Hsp60 (cHsp60) antibody as a loading control.

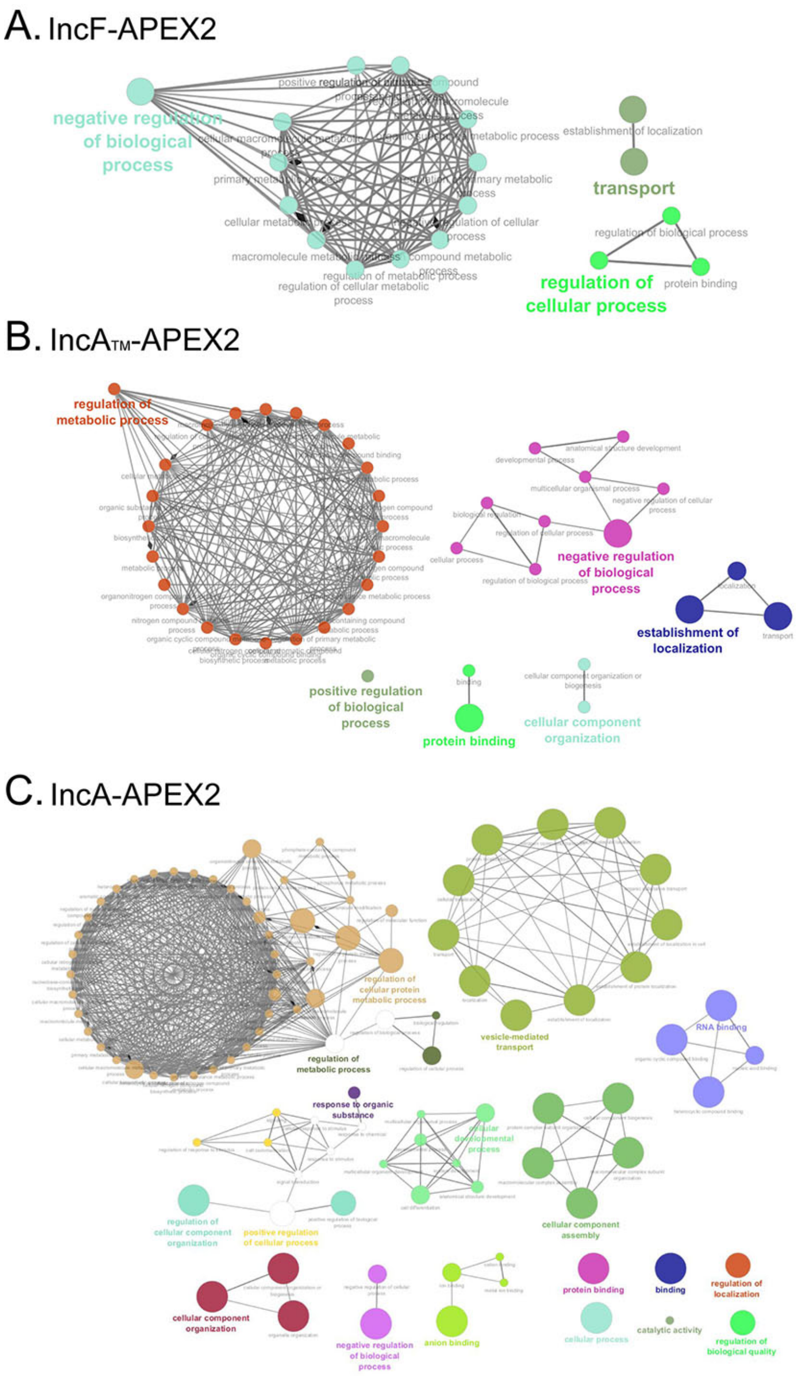

Fig S2. Visualization of global biological processes and molecular function of AP-MS identified statistically significant eukaryotic proteins using C. trachomatis L2 Inc-APEX2 transformants. Affinity purified-mass spectrometry (AP-MS) identified spectra were compared to the Homo sapiens database using Mascot and then

Significance Analysis of INTeractome (SAINT) was applied to identify statistically significant proteins (BFDR ≤ 0.05) from each dataset. Global networks identified using each C. trachomatis L2 transformed with (A) IncF-APEX2, (B) IncATM-APEX2, and (C) IncA-APEX2 are shown.

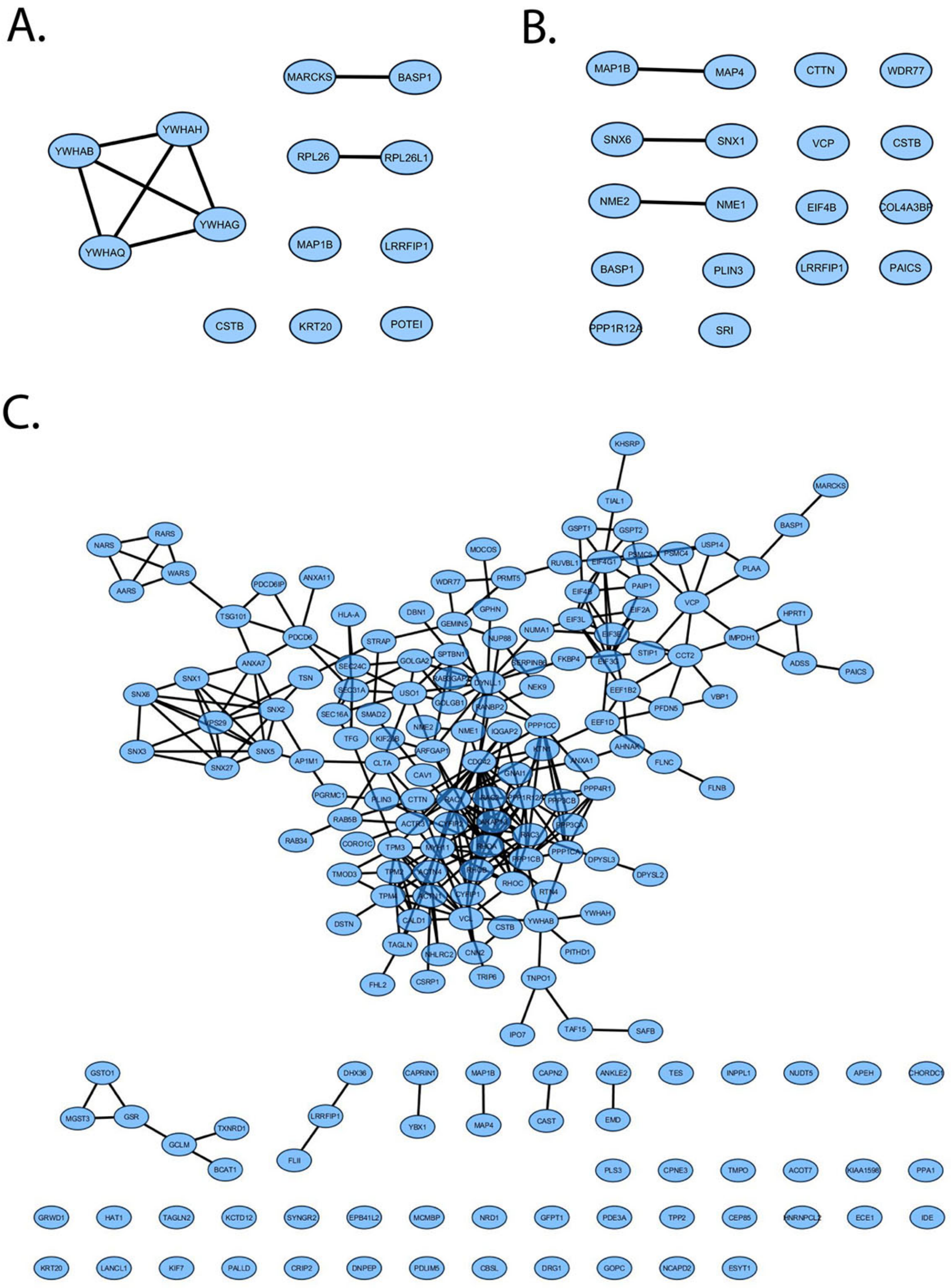

Fig S3. STRING network analysis of statistically significant eukaryotic proteins.

Significance Analysis of INTeractome (SAINT) was applied to identify statistically significant AP-MS identified eukaryotic proteins using C. trachomatis L2 IncF-APEX2,

IncATM-APEX2, and IncA-APEX2. STRING network (0.7 high confidence) visualization of eukaryotic proteins identified by mass spectrometry (SAINT BFDR ≤ 0.05) from each C. trachomatis L2 (A) IncF-APEX2, (B) IncATM-APEX2, and (C) IncA-APEX2.

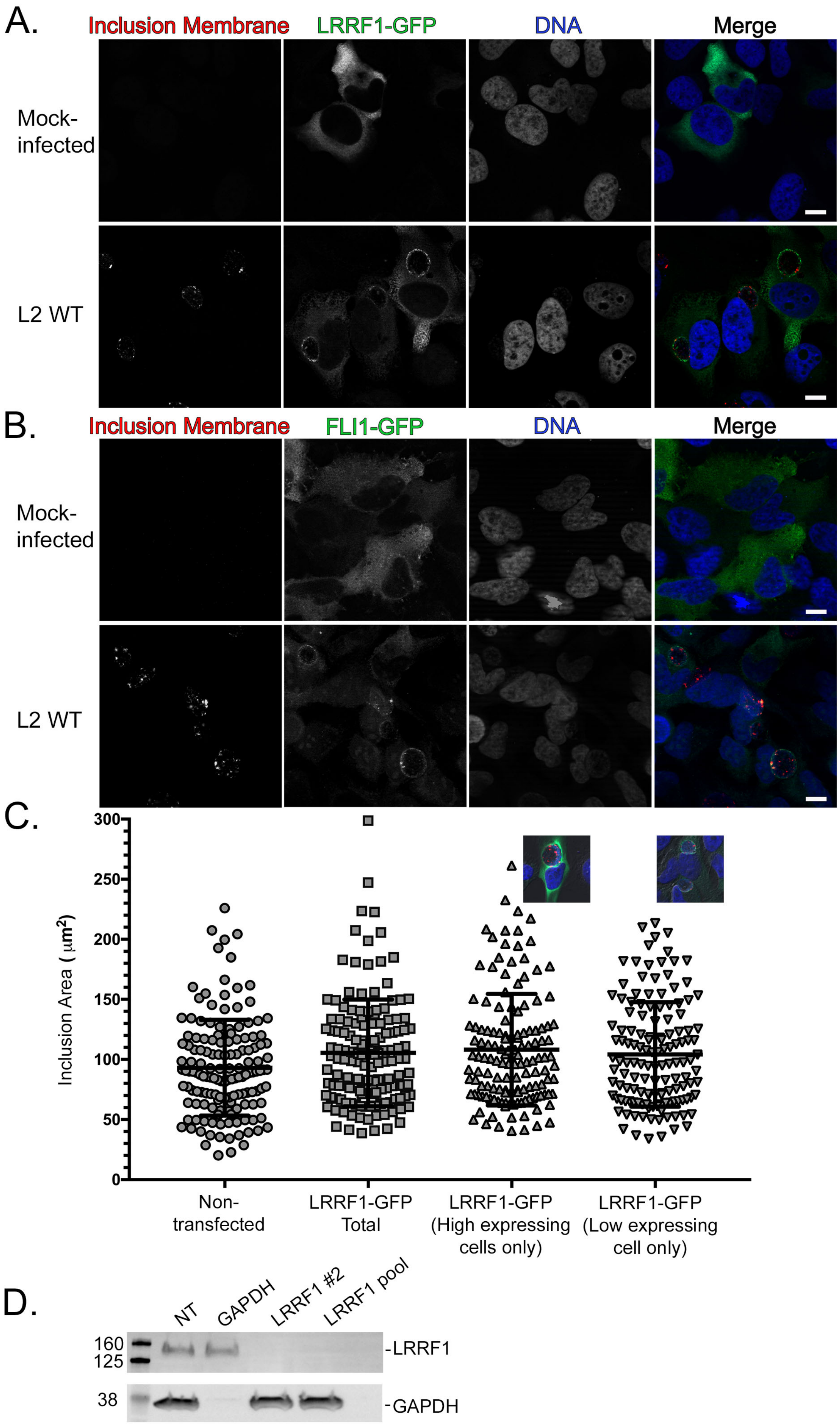

A.
Inclusion Membrane
LRRF1-GFP
DNA
Merge
Mock-infected
L2 WT
B.
Inclusion Membrane
FLI1-GFP
DNA
Merge
Mock-infected
L2 WT
C.
Inclusion Area (μm²)
300
250
200
150
100
50
0
Non-transfected
LRRF1-GFP Total
LRRF1-GFP (High expressing cells only)
LRRF1-GFP (Low expressing cell only)
D.
NT
GAPDH
LRRF1 #2
LRRF1 pool
160
125
38
-LRRF1
-GAPDH

Fig S4. The effect of LRRF1 -GFP and FLII-GFP overexpression and LRRF1 knockdown on *C. trachomatis* progeny production.

HeLa cells seeded onto coverslips were transfected with (A) 100 ng pCMV6-AC-LRRF1-GFP or (B) 500 ng pCMV6-AC-FLII-GFP. Transfected cells were either mock-infected or infected with *C. trachomatis* L2 wild-type at 6 hours post-transfection. At 24 hpi, HeLa cells were paraformaldehyde fixed, 0.5 % Triton X-100 permeabilized, and stained for immunofluorescence to visualize the inclusion membrane (CT223; red), DNA (DRAQ5; blue) and (A) LRRF1-GFP or (B) FLII-GFP. Coverslips were imaged using a Zeiss with ApoTome.2 at 100x. Scale bar = 10 µm. (C) Inclusion area measurements from HeLa cells transfected with pCMV6-AC-LRRF1-GFP and infected with *C. trachomatis* L2 wild-type (as above) were compared to non-transfected HeLa cells infected with *C. trachomatis* L2 wild-type. Inclusion area is reported for non-transfected, LRRF1-GFP Total (the inclusions from both high and low LRRF1-GFP expressing cells) and broken into LRRF1-GFP high and low expression only (see inset). Two independent experiments were performed. Inclusion area was graphed in GraphPad Prism 7 and a one-way ANOVA with Tukey's multiple comparisons post-hoc test was performed to determine statistical significance. There was no significant difference in inclusion area.

(D) siRNA knockdown of LRRF1 in HeLa cells. 20 nM of non-targeting (NT), GAPDH, LRRF1 single siRNA or 3 pooled siRNAs were reverse transfected as indicated into HeLa cells that were then infected with *C. trachomatis* L2 wild-type at 48 hours post-transfection and collected 24 hours later. Lysates from siRNA treated, *C. trachomatis* L2 wild-type infected cells were collected in 2x Laemmli sample buffer, electrophoresed, transferred to PVDF and blotted to confirm siRNA knockdown efficiency of LRRF1 and GAPDH.

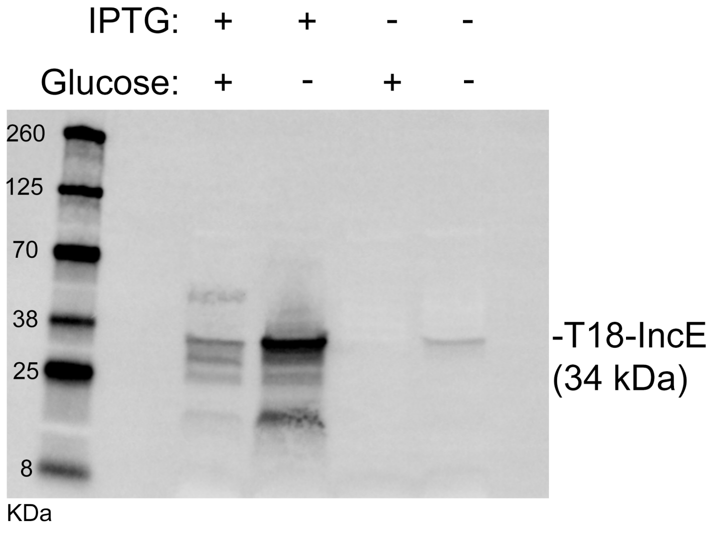

Fig S5. T18-IncE is expressed in *E. coli*

DH5α lacI[q] *E. coli* were transformed with pUT18C-IncE and grown overnight. Expression of T18-IncE was induced or not using 0.5 mM IPTG and with or without the presence of 0.4% glucose, then grown for 4 hours at 30°C. Bacterial lysates were collected, separated by SDS-PAGE, and transferred to a PVDF membrane. The membrane was blotted for expression of T18-IncE using an antibody against T18 (T18-IncE 34 kDa).

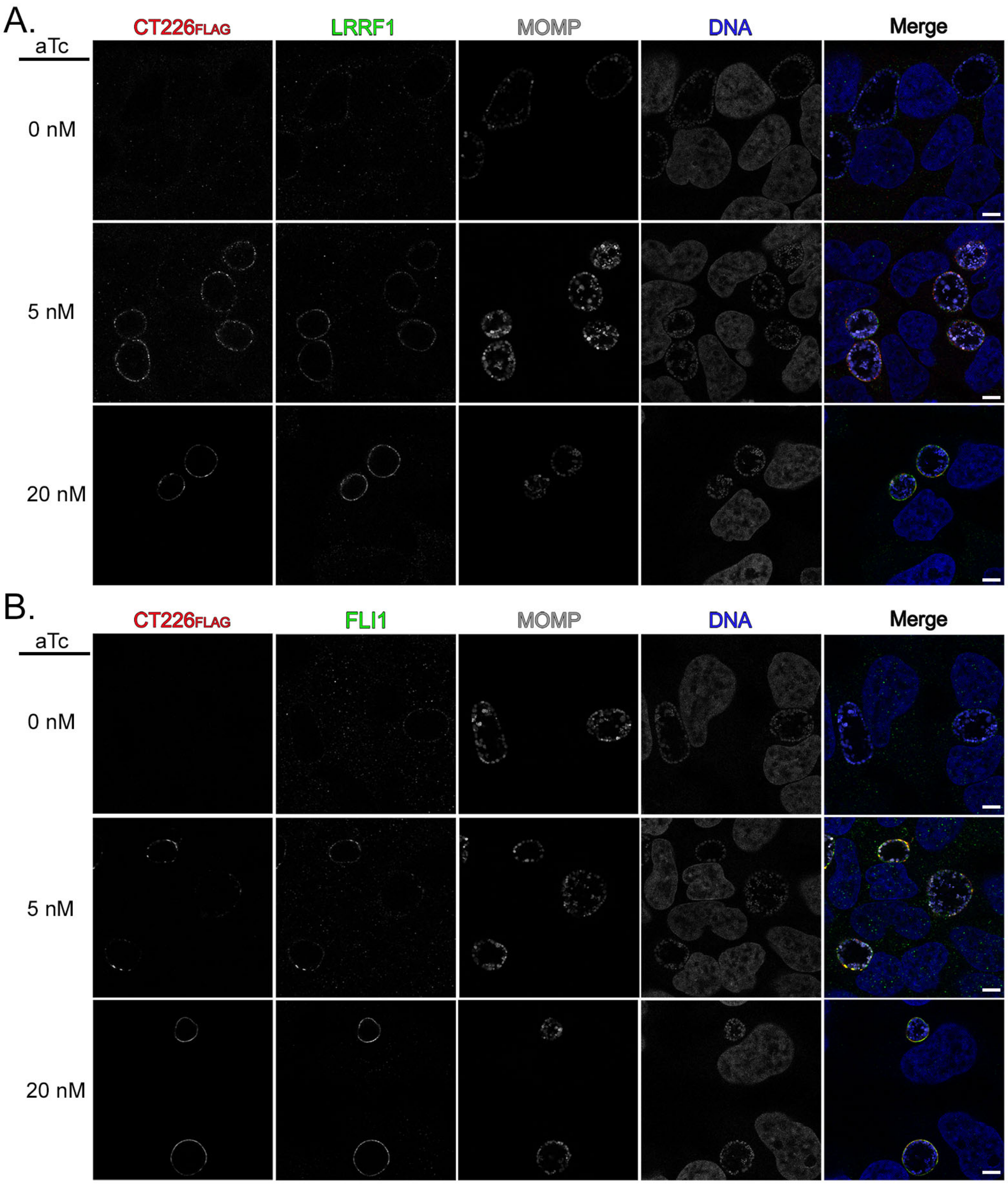

Fig S6. Overexpression of CT226FLAG from *C. trachomatis* L2 CT226FLAG transformants results in increased LRRF1 and FLII at the inclusion membrane HeLa cells seeded on glass coverslips were infected with C. trachomatis L2 CT226FLAG transformants and either not induced or induced for expression at 7 hpi using 5 nM or 20nM aTc. At 24 hpi, coverslips were fixed with 3% formaldehyde and 0.022% glutaraldehyde, permeabilized with methanol, and stained for immunofluorescence to visualize construct expression (FLAG; red), Chlamydiae (MOMP; gray), DNA (DAPI; blue), and A) LRRF1 (green) or B)

FLII (green). Coverslips were imaged using a Zeiss confocal LSM 800 with 63x magnification and 2x zoom. Scale bar = 5 μm. Images were captured using the same exposure time (set for 20 nM aTc images) for uninduced and 5 nM aTc samples.

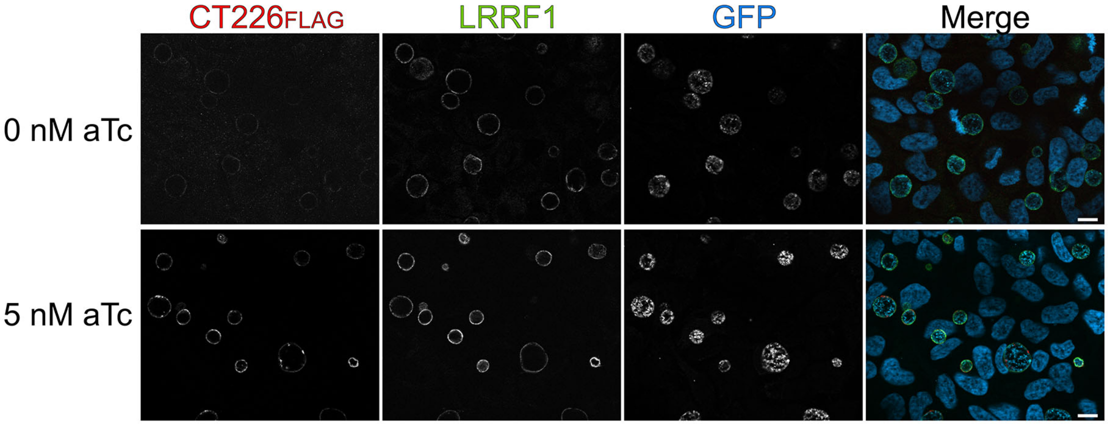

Fig S7. Assessment of CT226FLAG expression on LRRF1 2 localization using C. trachomatis L2 CT226FLAG infected HeLa cells using normal exposure levels

HeLa cells seeded on glass coverslips were infected with C. trachomatis L2 CT226FLAG transformants and either not induced or induced for expression at 7 hpi using 5 nM. At 24 hpi, coverslips were fixed with 4% paraformaldehyde, permeabilized with 0.5% triton X-100, and stained for immunofluorescence to visualize construct expression (FLAG; red), LRRF1 (green), GFP expressing Chlamydiae (pseudo-color blue), and DNA (DAPI; blue). Coverslips were imaged using a Zeiss with Apotome 2.1 with 100x magnification. Scale bar = 10 μm.

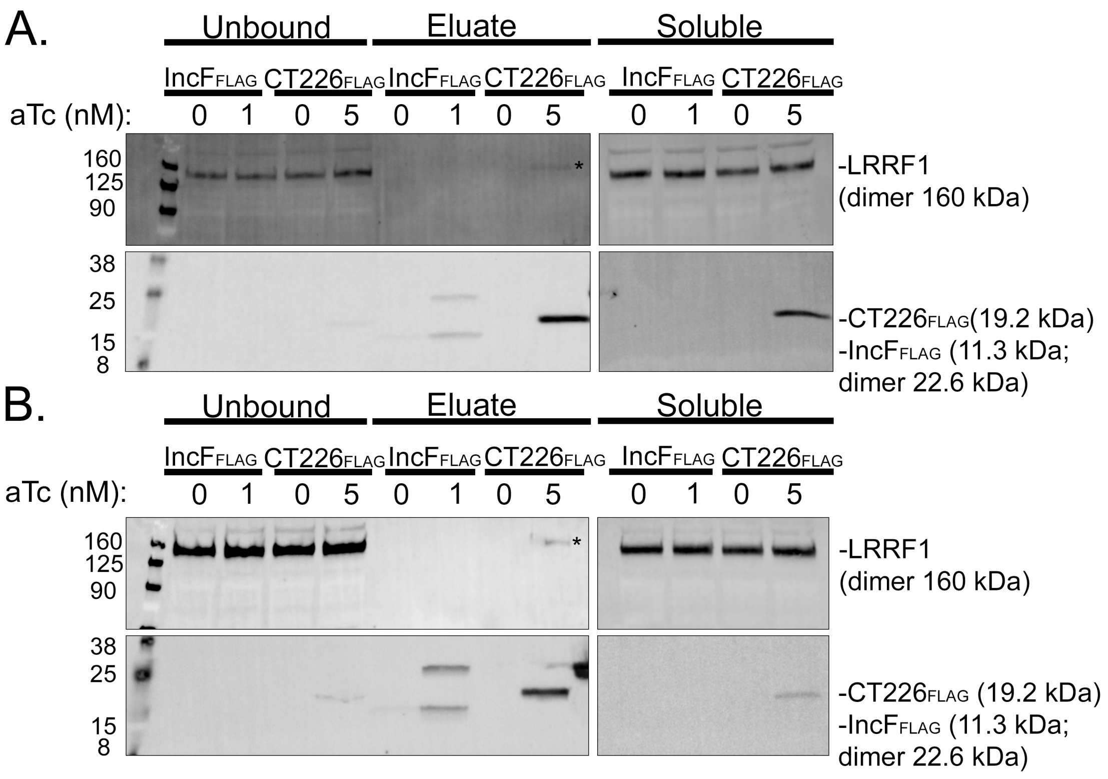

Fig S8. Co-immunoprecipitation of endogenous LRRF1 with *C. trachomatis* L2 CT226$_{FLAG}$

HeLa cells seeded in a 6-well plate with glass coverslips were infected with *C. trachomatis* L2 CT226$_{FLAG}$ or IncF$_{FLAG}$ and either not induced or induced for expression at 7 hpi with 5 nM aTc (CT226$_{FLAG}$) or 1 nM aTc (IncF$_{FLAG}$). At 24 hpi, cells were collected, solubilized, normalized, and affinity purified using FLAG beads. The clarified lysates (soluble), unbound fractions and eluates were probed for construct expression (FLAG; CT226$_{FLAG}$ 19.2 kDa; IncF$_{FLAG}$ 11.3 kDa monomer and 22.6 kDa dimer) and LRRF1 (dimer 160 kDa). (A) and (B) are representative of two biological replicates. A total of three independent experiments were performed.
